# At-Sea Distribution and Prey Selection of Antarctic Petrels and Commercial Krill Fisheries

**DOI:** 10.1371/journal.pone.0156968

**Published:** 2016-08-17

**Authors:** Sébastien Descamps, Arnaud Tarroux, Yves Cherel, Karine Delord, Olaf Rune Godø, Akiko Kato, Bjørn A. Krafft, Svein-Håkon Lorentsen, Yan Ropert-Coudert, Georg Skaret, Øystein Varpe

**Affiliations:** 1 Norwegian Polar Institute, Fram Centre, 9296 Tromsø, Norway; 2 Centre d’Etudes Biologiques de Chizé, UMR 7372 du CNRS-Université de La Rochelle, 79360 Villiers-en-Bois, France; 3 Institute of Marine Research, PO Box 1870 Nordnes, 5817 Bergen, Norway; 4 Norwegian Institute for Nature Research, 7485 Trondheim, Norway; 5 CNRS, UMR7178, 67037 Strasbourg, France; 6 Université de Strasbourg, IPHC, 23 rue Becquerel, 67087 Strasbourg, France; 7 University Centre in Svalbard, 9171 Longyearbyen, Norway; 8 Akvaplan-niva, Fram Centre, 9296 Tromsø, Norway; Liverpool John Moores University, UNITED KINGDOM

## Abstract

Commercial fisheries may impact marine ecosystems and affect populations of predators like seabirds. In the Southern Ocean, there is an extensive fishery for Antarctic krill *Euphausia superba* that is projected to increase further. Comparing distribution and prey selection of fishing operations versus predators is needed to predict fishery-related impacts on krill-dependent predators. In this context, it is important to consider not only predators breeding near the fishing grounds but also the ones breeding far away and that disperse during the non-breeding season where they may interact with fisheries. In this study, we first quantified the overlap between the distribution of the Antarctic krill fisheries and the distribution of a krill dependent seabird, the Antarctic petrel *Thalassoica antarctica*, during both the breeding and non-breeding season. We tracked birds from the world biggest Antarctic petrel colony (Svarthamaren, Dronning Maud Land), located >1000 km from the main fishing areas, during three consecutive seasons. The overall spatial overlap between krill fisheries and Antarctic petrels was limited but varied greatly among and within years, and was high in some periods during the non-breeding season. In a second step, we described the length frequency distribution of Antarctic krill consumed by Antarctic petrels, and compared this with results from fisheries, as well as from diet studies in other krill predators. Krill taken by Antarctic petrels did not differ in size from that taken by trawls or from krill taken by most Antarctic krill predators. Selectivity for specific Antarctic krill stages seems generally low in Antarctic predators. Overall, our results show that competition between Antarctic petrels and krill fisheries is currently likely negligible. However, if krill fisheries are to increase in the future, competition with the Antarctic petrel may occur, even with birds breeding thousands of kilometers away.

## Introduction

Through the last century, fisheries have reached levels that impact the abundance and structure of harvested stocks [1–3], as well as animals at higher trophic levels that rely on these stocks for foraging [4,5]. Marine predators such as seabirds play an essential role in the maintenance of ecosystem function (e.g. [6]) and may be affected by fisheries in different ways [4,5,7,8]. Fisheries can induce increased mortality rates in seabirds through by-catch [9–11]. They may also affect seabirds through competition when both rely on the same resource, and prey depletion by fisheries may increase competition among predators depending on the same resource [12]. Conversely, in some cases, seabirds may benefit from fisheries interactions through higher food availability in the form of discards [5,13,14,15].

Antarctic krill *Euphausia superba* is a pivotal species in the Southern Ocean food webs [16–18] and many top predators depend on krill as a food resource [19–24]. The Antarctic krill fishery was initiated in 1972 and is only authorized in specific areas (subareas 48.1 to 48.4, subarea 48.6 and divisions 58.4.1 and 58.4.2, [25]). Fishing is currently only conducted in some of these areas in the Scotia Sea, mainly between and around the South Orkneys, South Shetlands and South Georgia. Fishing vessels operate throughout most of the year using pelagic midwater trawls in the upper 250 m. The krill stock is still regarded as one of the world’s most under-exploited and the annual harvest levels are currently < 300,000 tons [26]. This is less than the catch limit set to 620,000 tons, which is considered to be precautionary, and far below the theoretical TAC (Total Allowable Catch Limit) of 5.6 million tons [25,27]. Due to the development of new harvesting and processing technologies, as well as an expansion in the range of products made from krill, krill fishery in the Southern Ocean is expected to increase [27]. In order to predict potential future impacts from such an increase on the population dynamics of krill-dependent predators, it is necessary to collect and compare distribution patterns of fishing operations versus predators [4]. Previous studies investigating the potential competition between krill fisheries and top predators focused on seals and penguins and generally only considered the breeding season [28,29–31]. Much less is known about flying and far-ranging seabirds as well as about the variation in the seabird-fisheries interactions throughout the year [32].

In this study, we first aimed at quantifying the overlap between the distribution of the main Antarctic krill fisheries activities and the distribution at sea of a flying krill-predator seabird, the Antarctic petrel *Thalassoica antarctica* [33]. The entire Antarctic petrel population has been estimated to be between 10 and 20 million individuals [34], suggesting that a minimum of 680,000 tons of Antarctic krill would be consumed per year by this species [33]. The Antarctic petrel relies on prey items available close to the surface [35] and searches large areas during single foraging trips (i.e., birds can travel as far as 2,000 km away from the colony during the breeding season; this study and [36]). We considered the distribution at sea, both during the breeding and non-breeding seasons, of individuals breeding at the world largest Antarctic petrel colony (Svarthamaren, Dronning Maud Land, 71°53’S, 5°10’E) and quantified the temporal variability in the overlap with krill fisheries. The Svarthamaren colony is located >1,000 km away from the krill fishing areas. However, considering the large at-sea movements of this species [36], spatial overlap between Antarctic petrel foraging areas and krill fisheries is highly plausible as both likely target areas of high krill abundance. This might be especially true during the non-breeding season when most of the commercial krill fishing occurs and when petrels are no longer central place foragers and can freely disperse at sea.

Moreover, besides examining potential overlap in spatial distribution, to understand the potential competition between different users of the same resource, we need to determine whether the same segments of the prey population (e.g. juveniles or adults) are targeted [37]. Therefore, in a second step, we studied the size frequency distribution (a proxy of the development stage) of Antarctic krill consumed by Antarctic petrels. By collating published data, we compared this information with what is known from other Antarctic krill consumers, including seabirds, sea mammals, and finally with commercial krill fisheries.

## Methods

### Ethics statement

Fieldwork (including logger deployments on Antarctic petrels and stomach content sampling) has been approved by the Norwegian Animal Research Authority (permits #3714 and 7935). Collection of data and sampling methods are detailed in the following sections.

### Antarctic petrel

The Antarctic petrel is one of several abundant seabird species of the Southern Ocean belonging to the order Procellariformes. It is a medium-sized petrel weighing *ca*. 600 g that lay one egg in late November / early December when the adjacent ocean is still heavily covered with sea ice. The incubation is shared by both parents and each incubation shift lasts for one to three weeks [38]. After hatching (mid January), the chick is guarded for another two weeks [38]. In this period, foraging trips gradually shorten until the chick is left unattended for the first time (end of January). From this point, both parents feed their chick until fledging at 6–7 weeks of age (early March). At Svarthamaren, the most important prey brought back to the chick is the Antarctic krill (this study and [33]). Outside the breeding season, the diet of Antarctic petrels is unknown but stable isotope analyses suggest that crustaceans also represent a substantial part (S1 Table). In other Antarctic petrel colonies or in Antarctic petrels sampled at sea, Antarctic krill also generally represents an important prey [39,40] but with some variation [41]. Myctophid fish are also important prey for Antarctic petrels and, in some years and/or places, may be the main ones by mass [41,42].

Antarctic petrels were captured between December and February in breeding seasons 2011/12, 2012/13 and 2013/14 at the Svarthamaren colony [34,43]. This colony is located *ca*. 200 km inland and hosts around 200,000 pairs of Antarctic petrels [44]. Breeding adults were captured (by hand or with a nylon loop attached at the end of a small fishing rode) on their nest during incubation or chick rearing, and instrumented with Global Positioning System (GPS) loggers (CatTrack 1, Catnip Technologies Ltd., Anderson, USA) just before leaving on a foraging trip. The original plastic packaging was replaced by waterproof heat-shrink tube, and the GPS units, weighing 18–20 g (ca. 3% of bird body mass), were taped to feathers (using Tesa^®^ tape; see S1 Text for details). We did not detect any detrimental effect of GPS loggers on foraging trip duration (S2 Text) or breeding success [45]. Birds were recaptured upon return to their nest (2 to 28 days after deployment) to retrieve the GPS units and download the data. GPSs recorded the locations of the birds along their foraging trip at intervals varying from 5 to 90 min (median = 10 min). The interval was set to record locations during the entire trip, considering both the GPS battery life expectancy (i.e. a higher location frequency being associated with a shorter life expectancy) and the expected duration of the trip (from several weeks in early incubation to just a few days in chick rearing, [38]). Over the three breeding seasons, a total of 133 foraging trips (from 124 individuals) were recorded, yielding >138,000 informative locations.

Outside the breeding season, at-sea distribution of Antarctic petrels was assessed using Global Location Sensors or GLS [46,47]. GLS (Biotrack MK4083 and Lotek LAT2500, weighing 2 and 3.5 g, respectively, i.e. < 1% of the bird body mass) were attached during the breeding season to a bird’s leg ring with a cable tie. GLS record light intensity for more than a year and thresholds in the light curves were used to determine daily sunrise and sunset. An internal clock allows for the estimation of the latitude based on day length and longitude based on the timing of local midday with respect to Universal Time [48]. While Biotrack loggers store raw light data, Lotek loggers summarise them on board and provide positions directly. Raw light data recorded by Biotrack GLS were analyzed following Philipps et al. [47]. Locations fixes were calculated from daylight data using BASTrak software (British Antarctic Survey, Cambridge, UK) using a light threshold of 4 and a sun elevation angle of -2. During *ca*. 2 week periods around the equinoxes (20–21 March and 22–23 September) and during the summer (November to February) when daylight is permanent (south of 66°S), latitude cannot be estimated. Position accuracy is relatively low (ca. 180 km, [47,49]) but GLS data are suitable to describe seabird distribution at large spatiotemporal scales, such as for oceanic species during winter. In our study, we deployed 46 Lat2500 (30 in 2011/12 and 16 in 2012/13) and 40 MK4083 loggers (all in 2012/13), and retrieved a total of 69 loggers (80%): 41 LAT2500 (21 in 2012/13 and 20 in 2013/14) and 28 MK4083 (in 2013/14). In total, 64 loggers functioned correctly (all LAT2500 and 23 out of 28 MK4083) and were used in this study.

### Antarctic krill

The Antarctic krill is a highly abundant euphausiid crustacean, distributed throughout the Southern Ocean with some regional variations [50]. It is a relatively long-lived, iteroparous macro-zooplankter with a total length of up to 60 mm [51]. Swarming is a central element of its behavior and a trait of relevance for predator-prey interactions, as well as interactions with fisheries. Antarctic krill spawns in spring and summer and lays consecutive batches of up to 1000 eggs [51]. It feeds primarily on phytoplankton and secondarily on protozoans and copepods [52].

In years 2011–2013, fishing of Antarctic krill was concentrated around South Georgia (subarea 48.3), and the South Orkney (subarea 48.2) and South Shetland (subarea 48.1) Islands, in areas located >2000 km from the Svarthamaren petrel colony (see Results). We obtained data on krill fishing activities for the years 2011 to 2013 from the Commission for the Conservation of Antarctic Marine Living Resource or CCAMLR [25]. The catches are reported on a haul-by-haul basis for conventional trawlers and every two hours for continuous trawlers, and summed up to a total of 31,473 trawl hauls. Data from October to December were removed because fishing effort was generally reduced or nil (S1 Fig) and very few petrel tracking data were available for that period (n = 12 tracks between end of November and end of December).

### Size of krill consumed by Antarctic petrels

In late January/early February 2013, we collected stomach contents by stomach lavage from 23 provisioning adult Antarctic petrels for prey characteristic and taxonomic identification of content [53]. Collection took place immediately after the return of the bird from a foraging trip and before they started feeding their chick. The 23 sampled birds were not fitted with a GPS and consequently their foraging areas were unknown. This stomach sampling means that chicks from sampled adults missed one meal and thus fast an extra 1–2 days. Indeed, both parents feed the chick and foraging trip duration last less than 4 days in late January/early February [38]. In petrels and albatrosses, chicks can easily miss 1 to 3 meals without any adverse effect on their growth or survival [54,55]. Consequently, this stomach sampling method was expected to have no or limited adverse effect on chicks from sampled Antarctic petrels. Unfortunately, no data were available to assess these potential effects.

Stomach contents were immediately frozen and later transferred to our laboratory for taxonomic analysis, following Cherel & Ridoux [56] and Cherel et al. [57]. Prey was identified using published keys and descriptions and by comparison with material held in our own reference collection [58–60]. Specifically, fish prey were identified from the morphology of otoliths and of distinctive bones (e.g. dentaries, vertebrae). Digested *Euphausia* species were determined by their typical round eyes, while antennular lappets and rostrum shape allowed identifying Antarctic krill from ice krill *Euphausia crystallorophias* [61]. Body length of Antarctic krill was assessed by measuring eye diameters and converting these to measurements of total length (TL) using the regression provided by Morris et al. [62]. TL was estimated from krill individuals subsampled from each stomach content sample. An average of 45 individual krill were subsampled per stomach content (range 2–70); these individuals were randomly chosen among all individual krill present in the sample.

### Size of Antarctic krill harvested by predators and trawls

We performed a review of published studies on the body length of Antarctic krill consumed by other predators (including fisheries). We searched, using both *Web of Science* and *Google Scholar*, different combinations of the following key words “Antarctic krill”, “content”, “scat”, “seal”, “seabird”, “whale”, “penguin”, “albatross”, “petrel”, “prion”, “fulmar”, “length”, or “size”. We found a total of 54 references, corresponding to 134 averages (and 77 modes) of krill total length consumed by Antarctic predators (S2 Table). We found only three references mentioning the size of krill consumed by whales [63–65]. Two of these studies were based on the size of krill available in whale foraging areas and not on the actual size of krill consumed [63,65]. These two references were not included in our quantitative analyses. Ten of those studies had sampled krill using trawls in the predator foraging areas (giving 11 estimates of average total length, and 14 estimates of modal length, from scientific trawls) or refer to results from commercial fishing (1 estimate of average total length, and 2 estimates of modal length). We also added data from CCAMLR [25] on the length of Antarctic krill harvested by fisheries for years 2009–2014, for each season (summer and winter) and krill fishing areas (48.1, 48.2 and 48.3; n = 28 additional estimates of average total length).

### Statistical methods

All analyses were done in R 3.1.1 [66]. For each year and month, we quantified the proportion of krill fishing area (kernel 95%) that overlapped with the Antarctic petrel distribution. To estimate petrel distribution, we considered three different levels: 30% (core areas–high intensity of use), 60% (intermediate intensity of use) and 95% (almost whole area) kernel utilization distribution (hereafter kernel UD). This choice allowed us to compare areas of contrasting level of utilization. In order to produce comparable kernel UDs, we used the same smoothing factor (h) for GLS and GPS location data. The smoothing factor was determined based on the average locational error attributed to GLS data (h = 150 km), which is typically much coarser than that of GPS data. Cell size for the output UDs was 1000 m, i.e. much finer than the scale of the geographic area covered. We used package *proj4* v.1.0–8 [67] for the projection of GPS and GLS coordinates and all map layers. We used package *adehabitatHR* v.0.4.13 [68] for the calculation of kernel UDs.

To analyze variations in krill size consumed by different predators and harvested by fisheries, we performed linear models (ANOVAs) with krill total length as the dependent variable. We first tested for a difference between the size of krill consumed by the different predator species. Then we compared the size of krill harvested by fisheries (commercial and scientific) and by marine birds/mammals during the winter and summer. Using linear mixed models with species included as a random effect (to take into account potential non-independence in our data due to repeated measurements on the same species) led to the same results (analyses done with the *lmer()* function from package *lme4*). We therefore only presented results from simple linear models. We used the *lm()* function from package *stats*.

## Results

### Distribution of Antarctic petrels and overlap with krill fisheries

**Fig 1:**
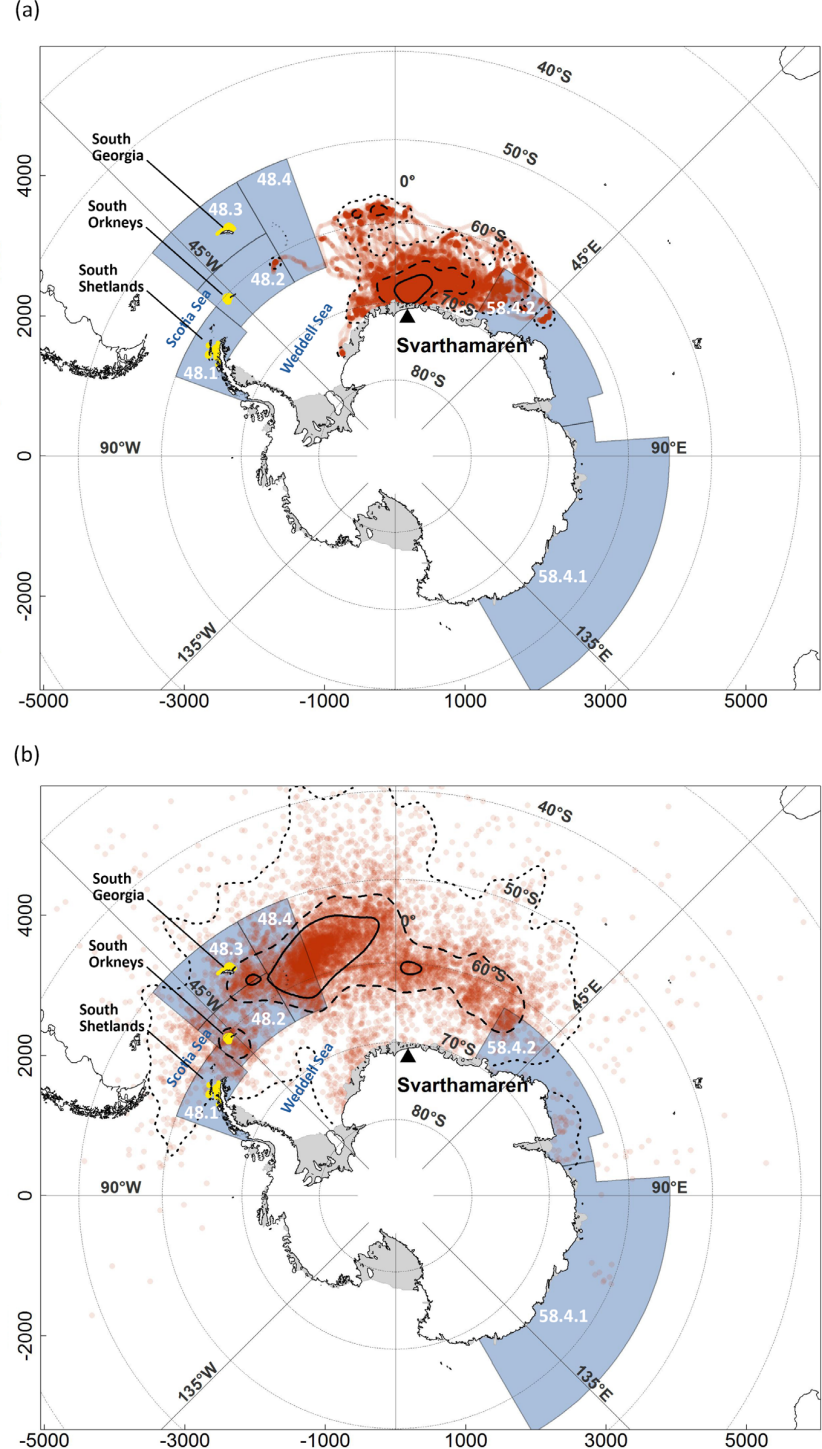
**Summer (a) and winter (b) distribution of Antarctic petrels breeding at Svarthamaren (71°53’S, 5°10’E).** The summer distribution was derived from locations pooled over December to February over 3 years, 2012–2014 (from GPS tracking); winter distribution derived from locations pooled over March to September and over 2 years (2012 and 2013; from GLS tracking). Continuous, dashed, and dotted lines show the 30, 60, and 95% kernel Utilization Distributions, respectively. The blue shaded area represents the zones where Antarctic krill fishing is permitted (numbers refers to CCAMLR sub-areas), and the yellow areas show where Antarctic krill fisheries occurred in years 2011–2014. Map projection is South Polar Stereographic, and the coordinates on both axes are in km.

The overall distribution area of Antarctic petrels differed greatly between summer (Fig 1A) and winter (Fig 1B). In summer the 95% kernel UD pooled over the three consecutive breeding seasons covered *ca*. 2.8 million km^2^ (Fig 1A). The 95% kernel UD in winter covered a much wider area (*ca*. 20.9 million km^2^), partly due to the imprecision in GLS positioning.

During the breeding season (December-February), Antarctic petrels did not forage in the fishing areas (Fig 1A), although one individual foraged once as far as area 48.2 (>2000 km from the colony). Consequently, there was no overlap between krill fisheries and the foraging areas of breeding Antarctic petrels.

**Fig 2:**
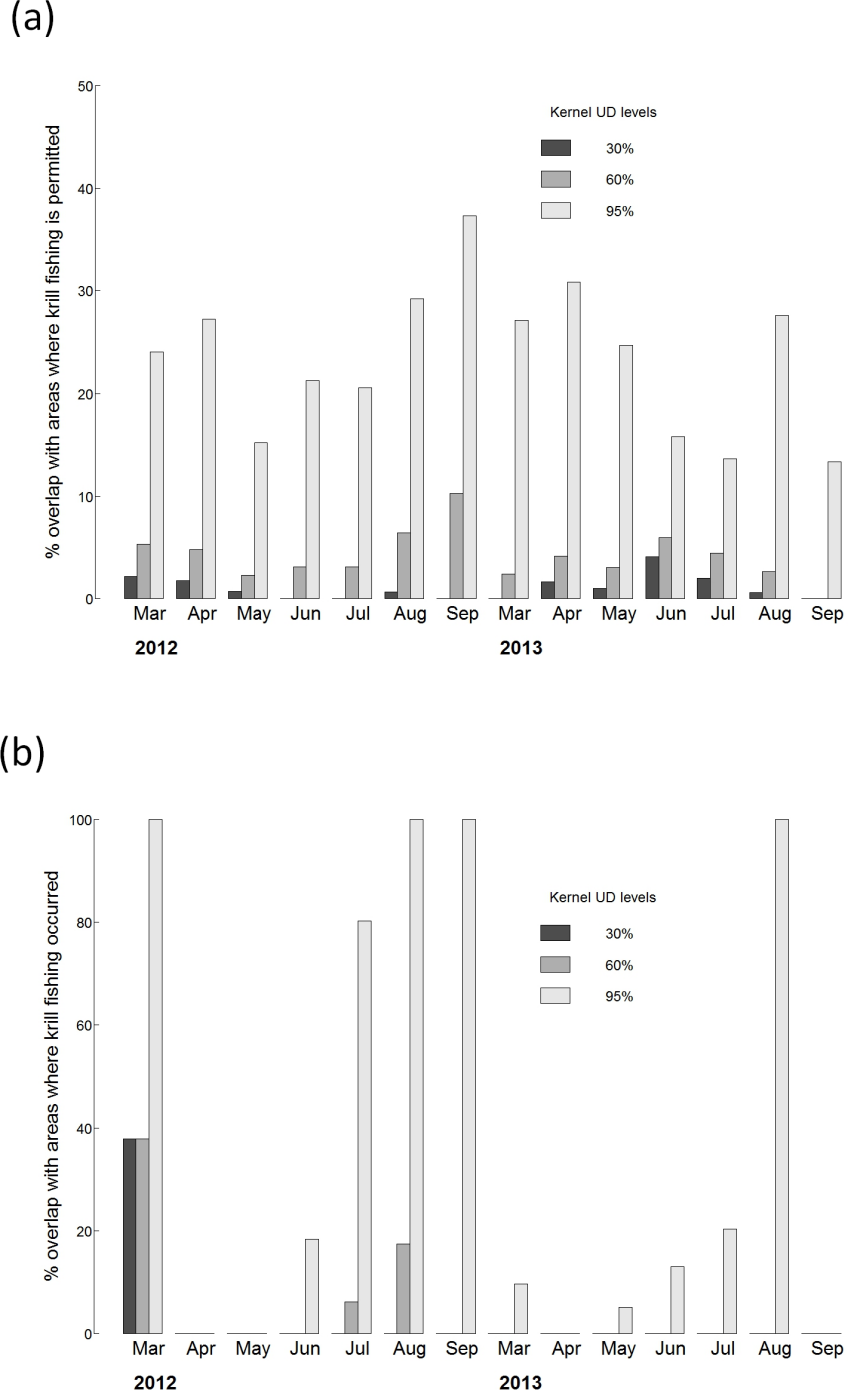
Monthly overlap between krill fishing areas and Antarctic petrel at-sea distribution (kernel Utilization Distribution) during two consecutive years. Only the non breeding season is shown here (overlap is nil during the breeding season). (a) represents the overlap with areas where krill fishing is permitted (i.e. with CCAMLR sub-areas 48.1 to 48.4, 58.4.1 and 58.4.2) and (b) the overlap with areas where krill fishing currently occurs.

During the non-breeding season (March-September), Antarctic petrel distribution encompassed a large part of the area where krill fishing is permitted (Fig 1B and Fig 2). The overlap between Antarctic petrel whole distribution (95% kernel) and CCAMLR subareas 48 (48.1 to 48.4) and 58.4 (58.4.1 and 58.4.2) varied between 13% and 37% depending on the month and year (Fig 2A). When considering only the sub-area 48 (48.1 to 48.4), the overlap increased to 30 and 83%. Taking into account the actual areas where krill fishing occurred reduced the overlap that varied greatly among and within seasons (Figs 1B and 2B and S2 Fig). When considering the birds’ whole distribution during the non-breeding season (95% kernel), overlap occurred around the South Shetland, South Orkney or South Georgia Islands (Fig 2B and S2 Fig) for half of the observed months. When looking at the intermediate density area of Antarctic petrels at sea (60% kernel), there was some overlap with fisheries in March, July and August 2012 when petrels were located around the South Orkneys and South Georgia (Fig 2B and S2 Fig). When considering the high density core area of petrels (30% kernel), the overlap was nil except in March 2012 when petrels were located around the South Orkneys where a large proportion of krill fisheries occurred (Fig 2B and S2 Fig).

### Size of Antarctic krill harvested by Antarctic petrels and other Antarctic predators

**Fig 3:**
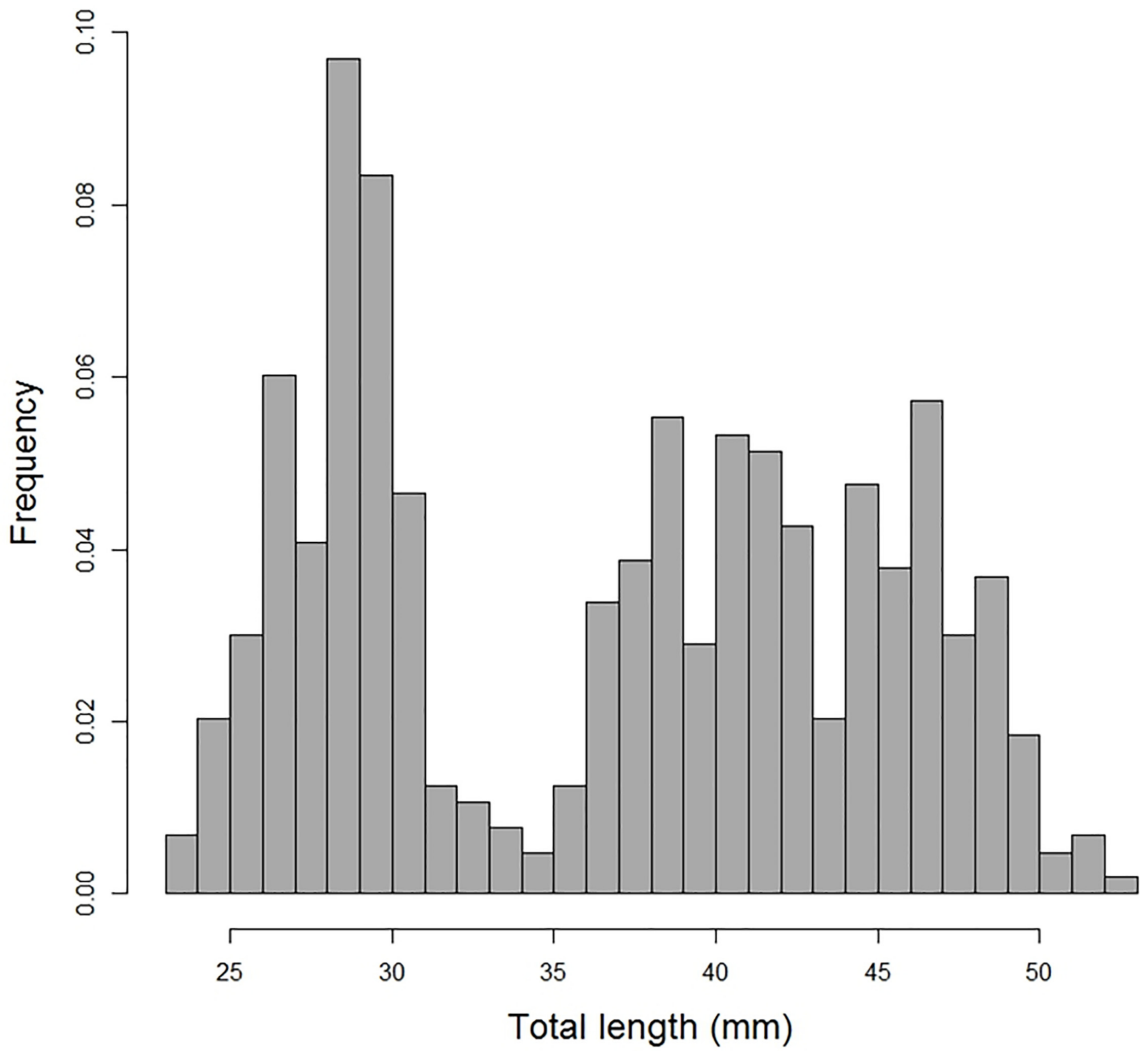
Size (total length)-frequency distribution of Antarctic krill harvested by Antarctic petrels in January/February 2014 (samples obtained at Svarthamaren, Dronning Maud Land).

**Fig 4:**
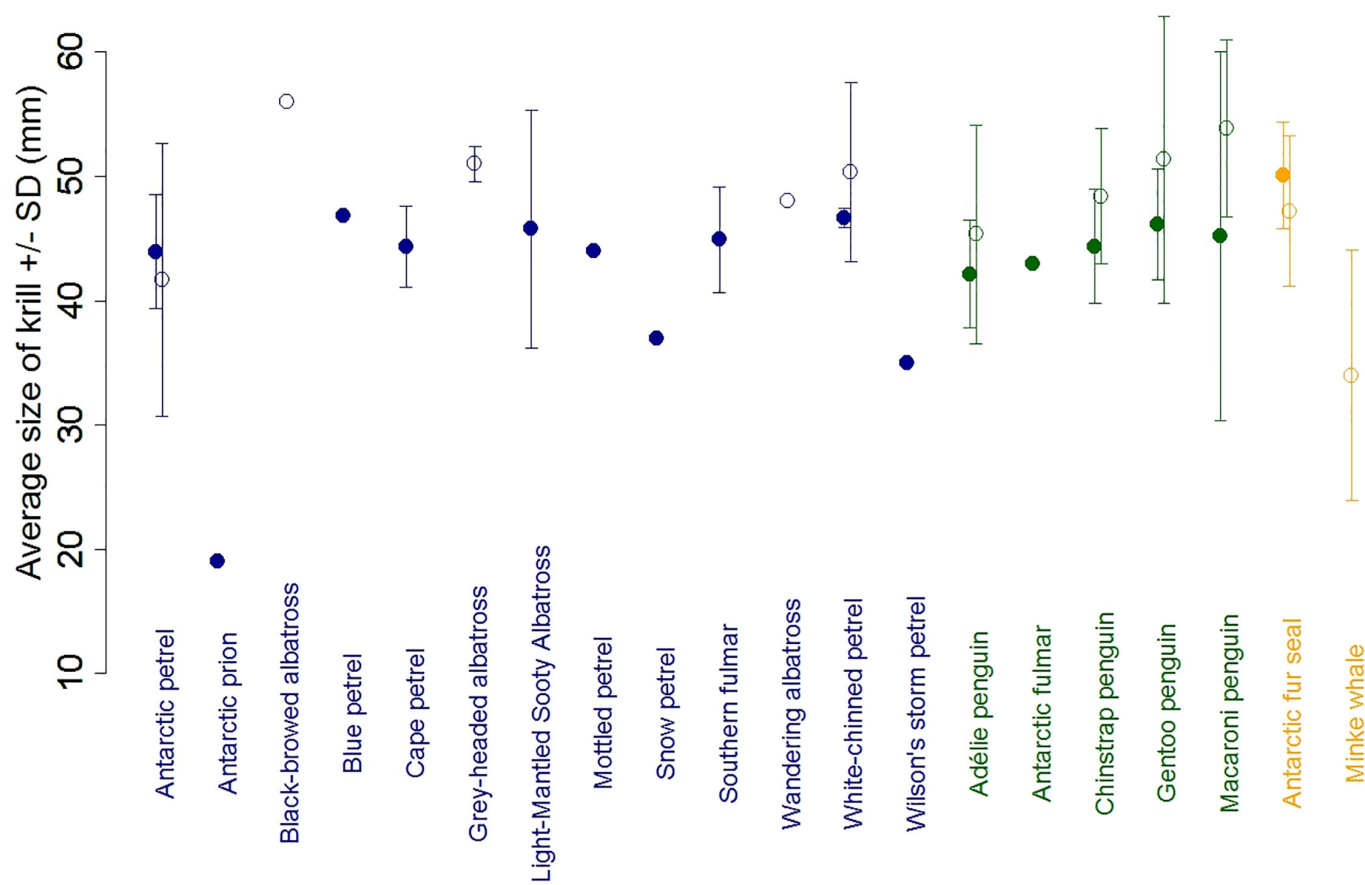
Average (±SD) size of Antarctic krill consumed by Antarctic predators. Blue colours correspond to surface-feeding seabirds, green to diving seabirds and orange to the Antarctic fur seal. Filled circles are estimates based on mean size of krill consumed and open circles are estimates based on modal size of krill consumed. Data are detailed in S2 Table.

In summer 2013, Antarctic petrel chicks at Svarthamaren were fed primarily with crustaceans (60% by mass), Antarctic krill being the dominant prey (98.7% of the total number of prey). Fish were the second most important prey by mass (35%; *Electrona antarctica*, *Notolepis coatsi* and *Pleuragramma antarcticum* being the most common fish species) but represented only 0.9% of the number of prey item. The total length of Antarctic krill consumed by Antarctic petrels averaged 37.2 mm but the distribution was bimodal with a clear mode at 30 mm and a less well-defined mode between 40 and 50 mm (Fig 3). This average size is among the lowest reported for all Antarctic seabirds and seals (Fig 4); 83% of the reported average size of krill consumed by Antarctic predators (birds and mammals) were ≥40 mm. There were significant variations in the average size of krill consumed by the different predators (F_19,114_ = 2.48, p = 0.002), but only driven by the Antarctic prion (n = 1 study) that consumed smaller krill than other species (Fig 4; p = 0.23 when the Antarctic prion is removed). This indicates that, on average, the size of krill consumed by Antarctic petrels did not differ from the one consumed by most Antarctic predators (Fig 4). There was no significant difference in prey size of diving versus surface-feeding predators (F_1, 132_ = 0.43, p = 0.51).

**Fig 5:**
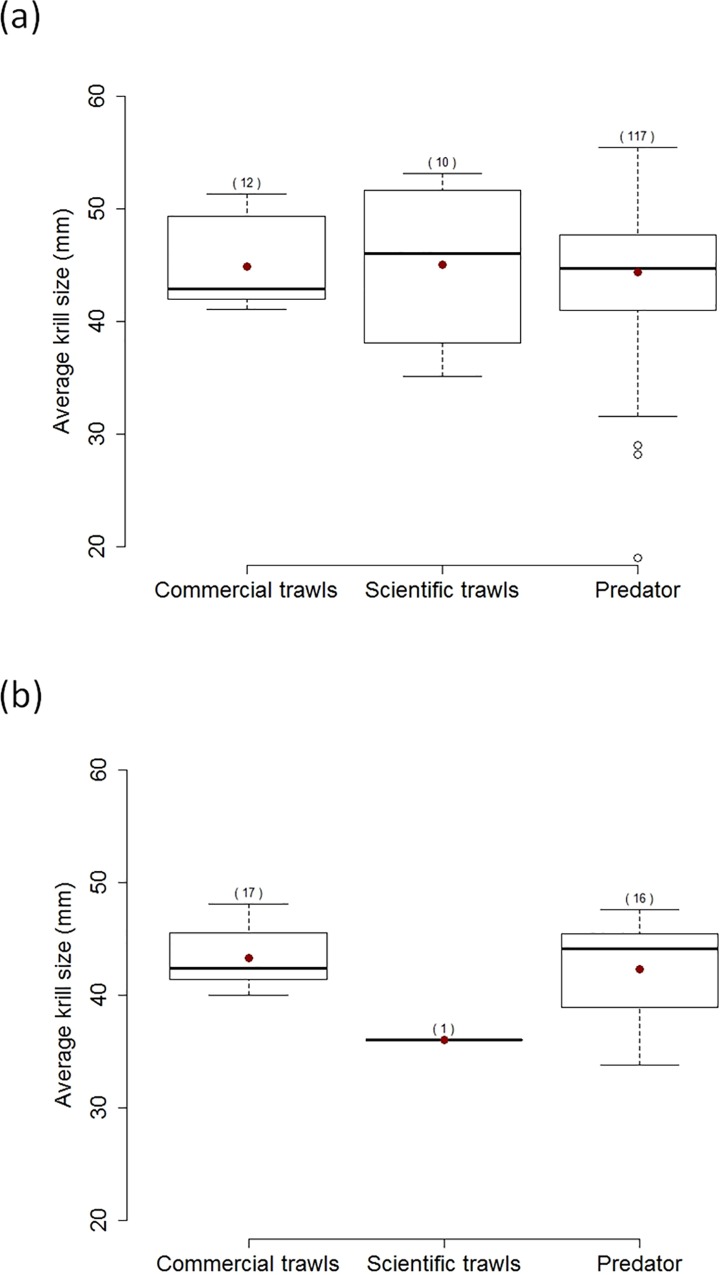
**Boxplots of the average size (total length) of Antarctic krill harvested by Antarctic predators (birds and mammals) and by scientific or commercial trawls in the summer ((a), December-March) and winter ((b), April-November).** Data are detailed in S2 Table. Red dots represent the mean values; sample sizes for each group are indicated in brackets.

Average krill size in scientific and commercial trawls did not differ from each other (F_1, 38_ = 0.016, p = 0.90) and from average size of krill consumed by seals and seabirds, neither during the summer (F_1, 137_ = 0.17, p = 0.68) nor the winter (F_1, 32_ = 0.20, p = 0.65; average krill size in trawls in the summer and winter season, respectively: 44.9 mm ± 5.3 SD and 42.9 ± 3.2 SD; average size of krill consumed by predators in the summer and winter season, respectively: 44.4 mm ± 5.7 SD and 42.3 ± 4.6 SD; Fig 5 and S3 Fig). Including year into the model (to take into account potential temporal variation in the size of krill harvested by predators or fisheries) did not change the results (p>0.6 in both summer and winter; S3 Fig).

## Discussion

### Spatial overlap between Antarctic petrel distribution at sea and Antarctic krill fisheries

Antarctic krill fisheries occur mostly around the Antarctic Peninsula, South Georgia and South Orkney Islands. Overall, those areas overlapped little with the distribution at sea of Antarctic petrels from Svarthamaren, and overlaps only occurred during the austral winter. During the breeding season (Dec-Feb), Antarctic petrels are constrained in their movements as they have to return regularly to the colony to incubate the egg or guard and feed the chick. Even if they travel very long distances during their foraging trips (up to 2000 km away from the colony), it is unlikely that they could reach the Scotia or North Weddell Seas without compromising their current reproduction. In summer, they were thus distributed east of the Weddell Sea and consequently did not utilize the commercial krill fishing grounds. Non-breeders may travel longer distances during the summer and potentially reach these krill fishing areas. Unfortunately, no data are currently available to test this hypothesis.

During the non-breeding season, petrels are not central-place foragers (i.e. they don’t have to return regularly to their nest) and can easily disperse in search of the most favorable feeding area. Petrels from Svarthamaren moved northwestward during the winter and were distributed in areas known to host very high krill densities [69]. Not surprisingly, these high krill density areas are also the ones targeted by krill fisheries so that the petrel whole distribution largely overlapped with areas where krill fishing is permitted, especially with sub-areas 48.1–48.4 (Fig 1B). However, Antarctic petrel spatial overlap with actual fisheries in winters 2012 and 2013 was limited, although high in some months. These results suggest that Antarctic petrels from Svarthamaren and fisheries may compete directly for krill but that this competition would only occur during the winter period with considerable inter-monthly and inter-annual variations. Antarctic petrels may also be attracted by fishing vessels and benefit from discards. However, this remains speculative, even if some previous at-sea observations indicate that Antarctic petrels may congregate around fishing vessels [70].

Getting fine-scale data on Antarctic petrel distribution outside the breeding season, combined with detailed information on their diet, would be needed to fully assess the interactions between potential krill fisheries and Antarctic petrels in the time windows when there is spatial overlap [71]. Yet, our results suggest that both krill fisheries and Antarctic petrels rely on the same krill stock during winter. Considering the small proportion of the krill standing stock taken by Antarctic petrels and commercial fisheries, current competition between petrels and fisheries is currently likely negligible. However, if krill fisheries are to increase in the future, our study indicates that competition with the Antarctic petrel may occur, even with birds breeding thousands of kilometers away.

### Is the Svarthamaren colony representative of the Antarctic petrel population?

Overlap with fisheries may be very different for Antarctic petrels breeding in the other colonies all around Antarctica and especially for petrels breeding closer to the western Weddell Sea or Antarctic Peninsula where most of the krill fishing occurs [34]. However, at-sea surveys indicate that Antarctic petrels are rare in the Antarctic krill fishing areas during the summer (November-March) and most studies report densities <0.04 Antarctic petrel / km^2^ around the Antarctic Peninsula, South Georgia and South Orkney Islands [72,73–78]. Extrapolating this petrel density (0.04) to the entire krill fishing area (sub-areas 48.1, 48.2 and 48.3; total surface of 2.525 millions of km^2^) would suggest that only ca. 100,000 Antarctic petrels (0.5–1% of the whole population, [34]) would forage in those areas during the summer.

The situation may be very different during the winter. The few studies that report seabird densities in the krill fishing areas during winter indicate that Antarctic petrel densities may be much higher than during the summer (e.g. up to 9.3 petrels / km2 in ice covered areas in the Scotia/Weddell Sea in July-August 1988, 5 Antarctic petrel / km2 around Elephant Islands in the South Shetlands, [79,80]). Antarctic petrels are, with snow petrels *Pagodroma nivea* and Adélie penguins *Pygoscelis adeliae*, the most numerous species observed during winter in krill fishing areas like the Scotia Sea [41] or South Shetlands [81]. An average density of 5 individuals per km^2^ would correspond to *ca*. 12 million Antarctic petrels foraging in the krill fishing areas outside the breeding season. This estimate, which would represent a very large proportion (>50%) of the entire Antarctic petrel population [34], is of course coarse but it exemplifies how the density of a krill predator may dramatically vary between seasons. This emphasizes the importance of considering the full annual cycle, including both the breeding and non-breeding seasons, when assessing the potential conflicts between fisheries and marine predators. And for efficient, long-ranging flyers such as petrels and albatrosses, it also stresses the need to consider birds breeding far away from the fishing grounds, when evaluating the potential conflicts between fisheries and bird foraging activities.

### Antarctic krill body size

In summer 2013, Antarctic petrels foraged on smaller krill, on average, than what has been reported in most previous studies on Antarctic seabirds and mammals (S2 Table). The small average size was due to a very high proportion of small krill individuals (<30 mm), which were likely juveniles (1 year olds). This does not necessarily imply that Antarctic petrels were targeting small krill but could rather indicate that small krill were highly abundant in the Antarctic petrel foraging areas. This could be due to high recruitment or size dependent vertical distribution patterns (e.g. larger individuals being underrepresented at the surface). Antarctic krill recruitment is highly variable from one year to the next so that the availability of small krill to predators also varies a lot among years [82–84]. Bimodal distributions of krill length in predator diets have indeed often been observed [41,64,85,86]. Our study provides interesting insights into krill biogeography and breeding biology, given the dominance in the diet of juvenile krill, and therefore presumably high abundance in the foraging areas of breeding Antarctic petrels from Svarthamaren.

Overall, we found very little evidence for a difference in krill size between predators and foraging tactics. Despite very large variation in their body size and weight (e.g. from ca. 200 grams for the blue petrel to >8000 grams for the wandering albatross), all petrel (including the Antarctic petrel), albatross and penguin species forage, on average, on Antarctic krill of the same size (Fig 5). Results on marine mammals also indicate that krill consumed by seals or whales has a similar size, on average, to krill consumed by seabirds (Fig 5). Moreover, we did not find any difference in krill size between krill consumed by predators and harvested by trawls (commercial or scientific; Fig 5 and S3 Fig). This does not mean that selection of particular krill stages or size may not occur [85,87]. However, this suggests that in general, most bird and mammal predators, as well as fisheries, seem to be mostly harvesting what is available in their environment and this varies in time and space. Some studies reported selective harvesting by seabirds or seals, with predators tending to feed on larger krill than caught in trawls [40,86]. However, opposite findings have also been reported and krill taken by predators may be smaller on average than krill caught in trawls [88]. Interpreting differences in the size of krill taken by predators and trawls should thus be done with caution, as krill size may vary even within a small geographical area (i.e. swarms separated by several hundred meters may have different size composition, [89]) and/or within a short time window (e.g. krill may grow up to 0.17 mm/day during the summer, [90]). As a consequence, as soon as trawl sampling is not done exactly at the same place, depth and time as predator foraging, comparison of krill size distributions may be misleading and results regarding potential selective harvesting should be taken with caution.

## Conclusions

Distribution of Antarctic petrels from Svarthamaren occasionally overlapped with krill fisheries during the non-breeding season. The level of overlap was generally low but varied greatly through time. Moreover, Antarctic petrels, as well as most Antarctic krill predators, target krill of similar size as the fisheries do. All these results indicate that competition, even if limited, may exist between Antarctic petrels and Antarctic krill fisheries. This emphasizes the importance of considering not only the breeding season and not only krill predators breeding near the fishing grounds when evaluating the potential conflicts between fisheries and bird foraging activities.

## Supporting Information

S1 Fig Temporal variation in monthly fishing effort of Antarctic Krill. (DOCX)

S2 Fig Monthly overlap between krill fishing areas and Antarctic petrel distribution. (DOCX)

S3 Fig Distribution of the average size of Antarctic krill harvested by Antarctic predators. (DOCX)

S1 Table Summary statistics for isotopic ratios of carbon (δ^13^C) and nitrogen (δ^15^N) measured in Antarctic petrel body feathers. (DOCX)

S2 Table Summary of the literature review. (DOCX)

S1 Text Retrieval rate of GPS loggers deployed on Antarctic petrels. (DOCX)

S2 Text Foraging trip duration of control and experimental (i.e. fitted with a GPS logger) Antarctic petrels. (DOCX)
