# Supplementary figures and images for: At-Sea Distribution and Prey Selection of Antarctic Petrels and Commercial Krill Fisheries
Source: PLoS One. 2016 Aug 17;11(8):e0156968. doi: 10.1371/journal.pone.0156968 (PMC4988635; doi:10.1371/journal.pone.0156968)

***S5 Figure***. Temporal variation in monthly fishing effort of Antarctic Krill in the period 2011-2013.


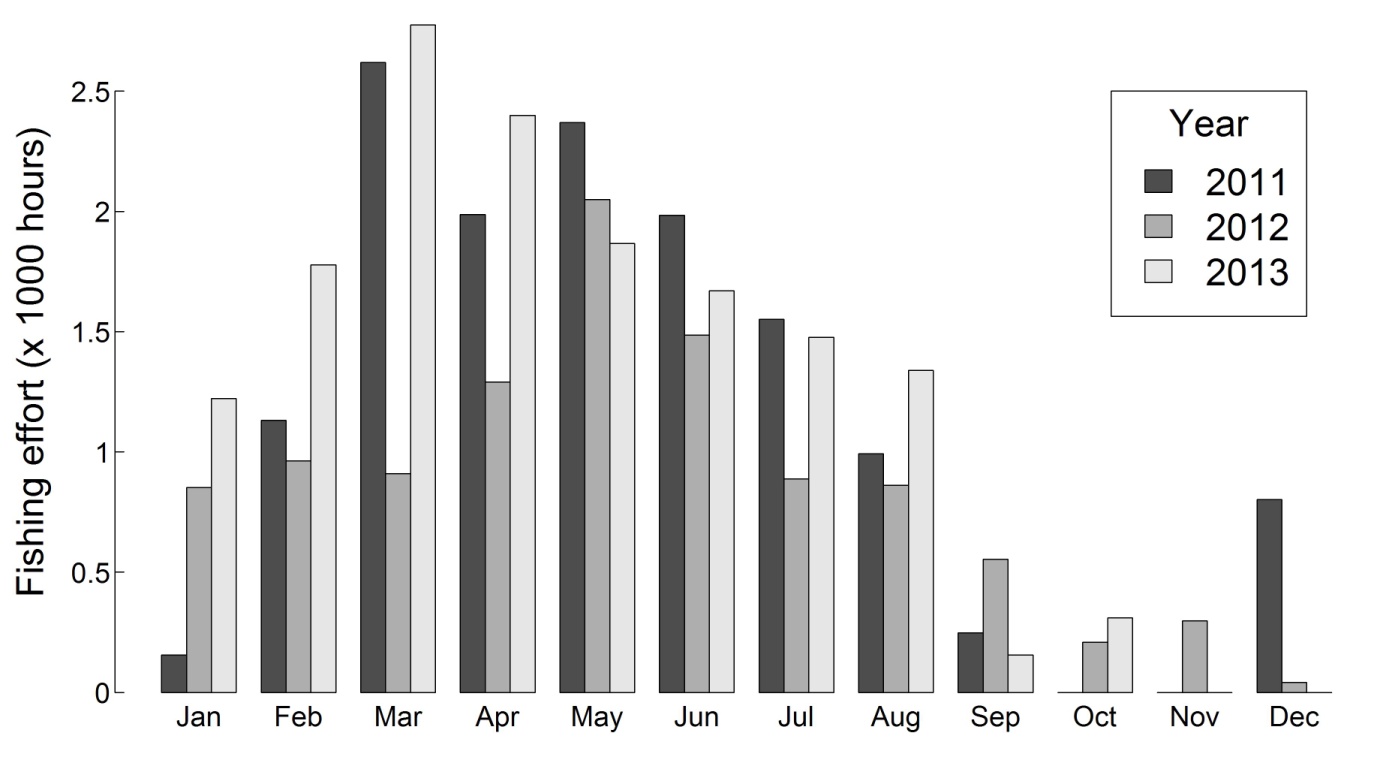

Supplement: S1 Fig — (DOCX) [file pone.0156968.s001.docx]
