# Supplementary material for: At-Sea Distribution and Prey Selection of Antarctic Petrels and Commercial Krill Fisheries
Source: PLoS One. 2016 Aug 17;11(8):e0156968. doi: 10.1371/journal.pone.0156968 (PMC4988635; doi:10.1371/journal.pone.0156968)
Supplement: S2 Fig — (DOCX) [file pone.0156968.s002.docx]

***S6 Figure***. *Monthly overlap between the krill fishing areas (blue: areas where krill fishing is permitted; yellow: areas where krill fishing occured in 2011-2013) and the Antarctic petrel distribution (30, 60, and 95% kernel Utilization Distributions are represented by continuous, dashed, and dotted lines respectively) within (a) and outside (b) the breeding season. Red dots show individual locations of Antarctic petrels during the corresponding months, i.e. GPS and GLS locations respectively for the breeding (a) and non-breeding (b) season. The blue shaded areas represent the zones where Antarctic krill fishing is permitted (numbers refers to CCAMLR sub-areas), and the yellow areas show where Antarctic krill fisheries occurred in years 2011-2014. Map projection is South Polar Stereographic.*

1. *Breeding season (2011/12, 2012/13 and 2013/14)*

*
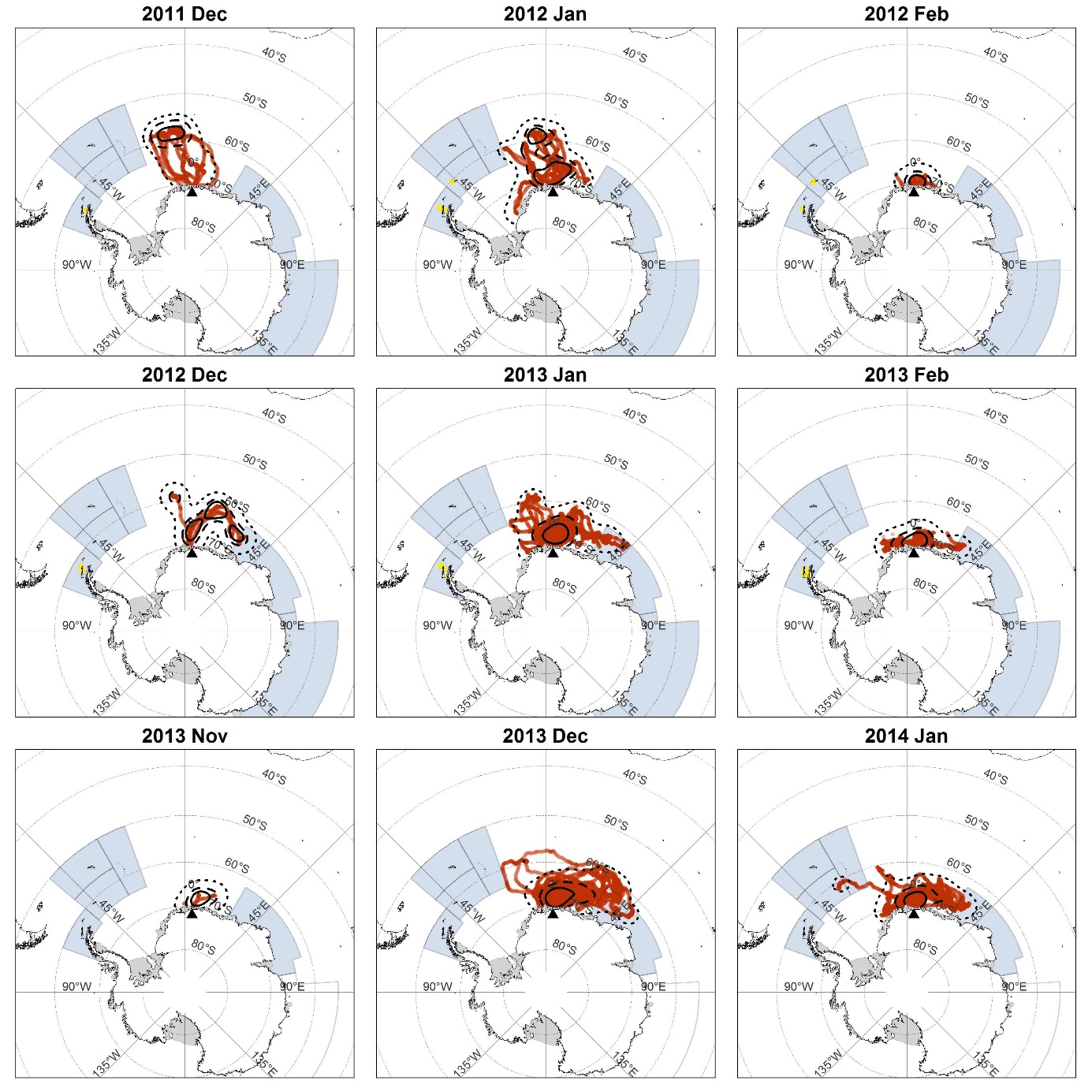
*

1. *Non-breeding season (2012 and 2013)*

*
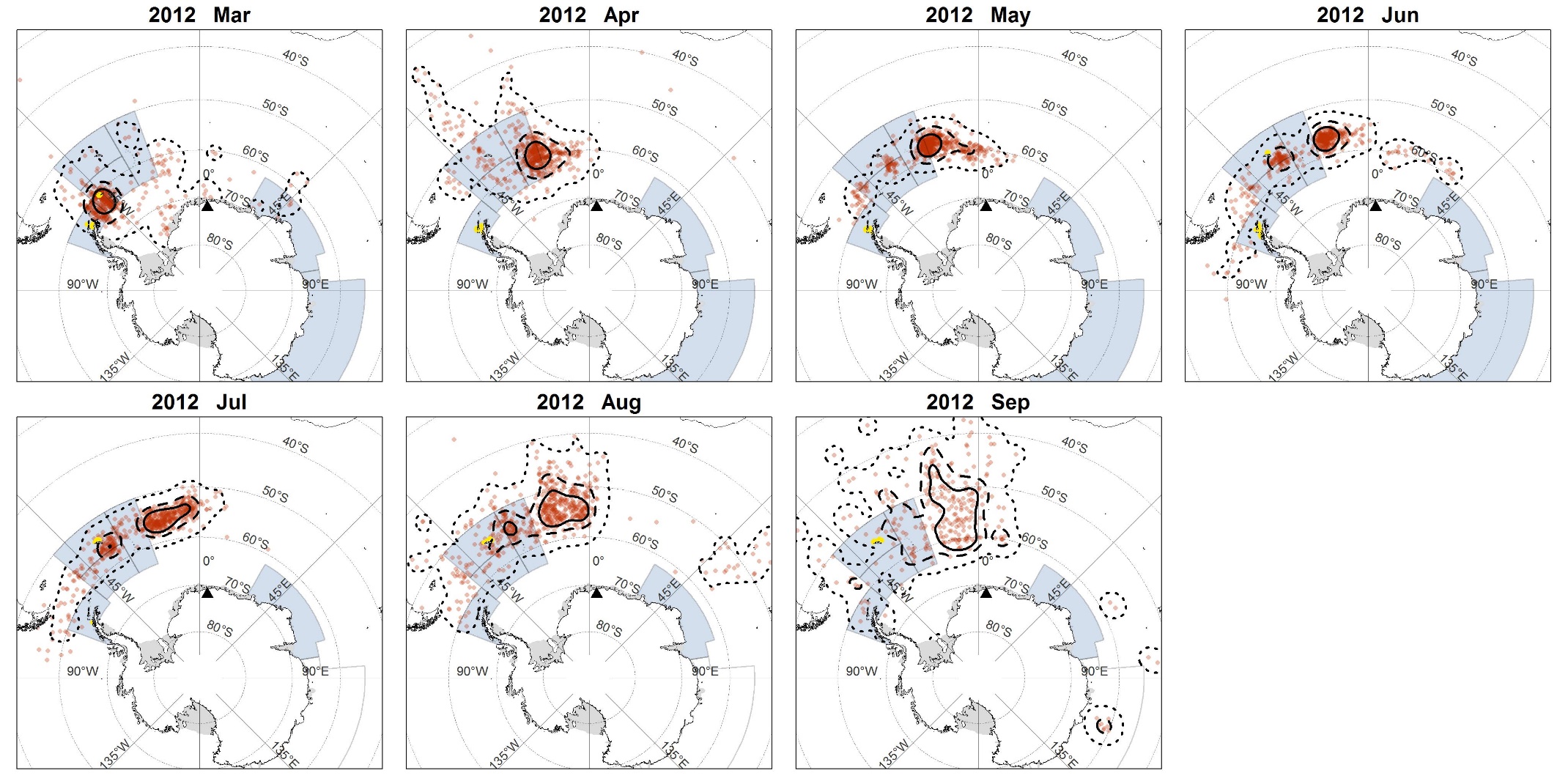
*

*
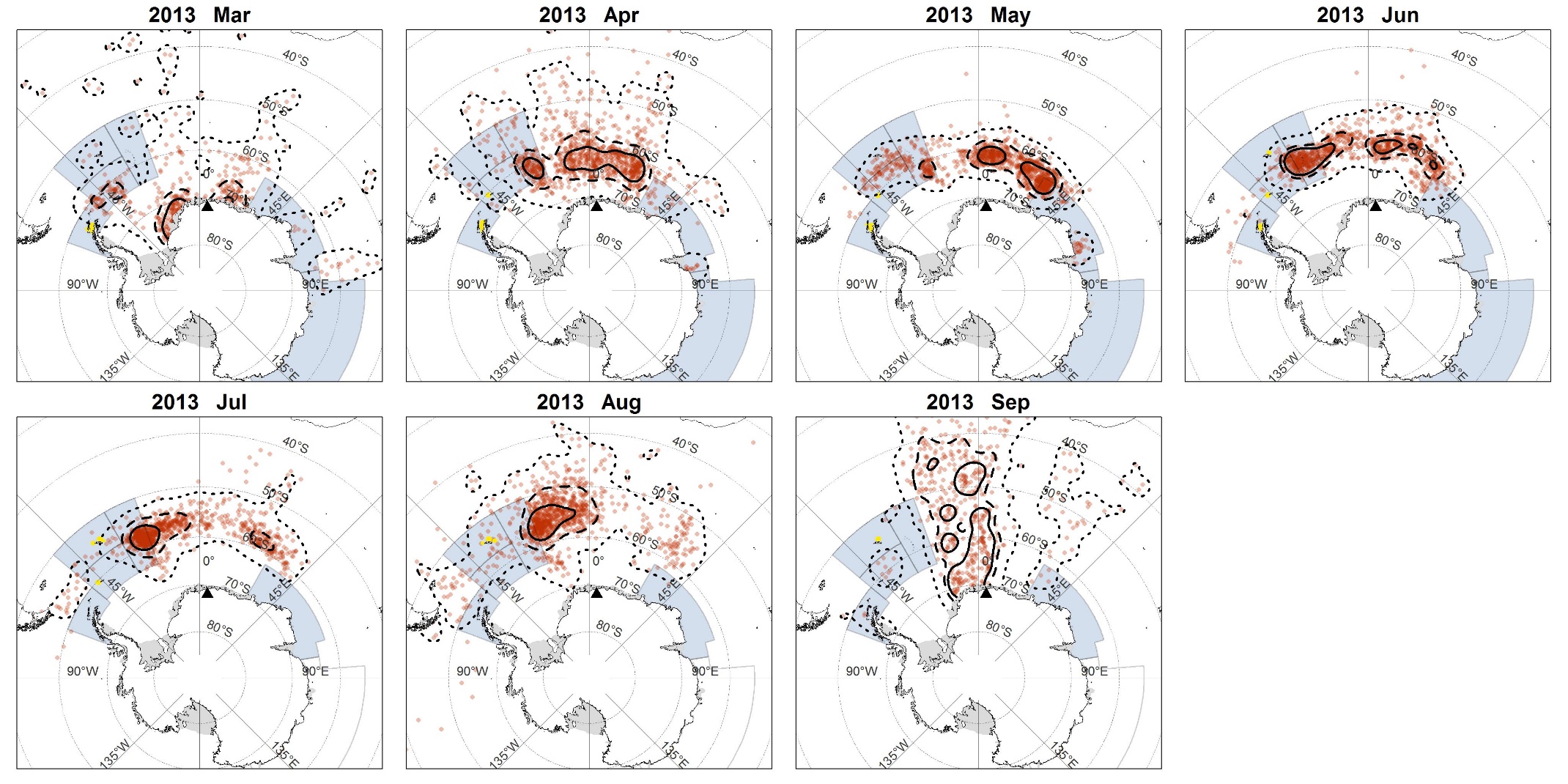
*
