# Supplementary material for: At-Sea Distribution and Prey Selection of Antarctic Petrels and Commercial Krill Fisheries
Source: PLoS One. 2016 Aug 17;11(8):e0156968. doi: 10.1371/journal.pone.0156968 (PMC4988635; doi:10.1371/journal.pone.0156968)

***S7 Figure***. (a) *Distribution of the* *average (grey) and modal (black) size of Antarctic krill harvested by marine predators (birds and mammals). Data are presented in Suppl. Mat. Table S1. (b) Average size of krill harvested by predators (black symbols) or trawls (commercial and scientific; white symbols). Only the scientific trawls performed as part of a study on seabirds or seals have been included.*

*(a)*


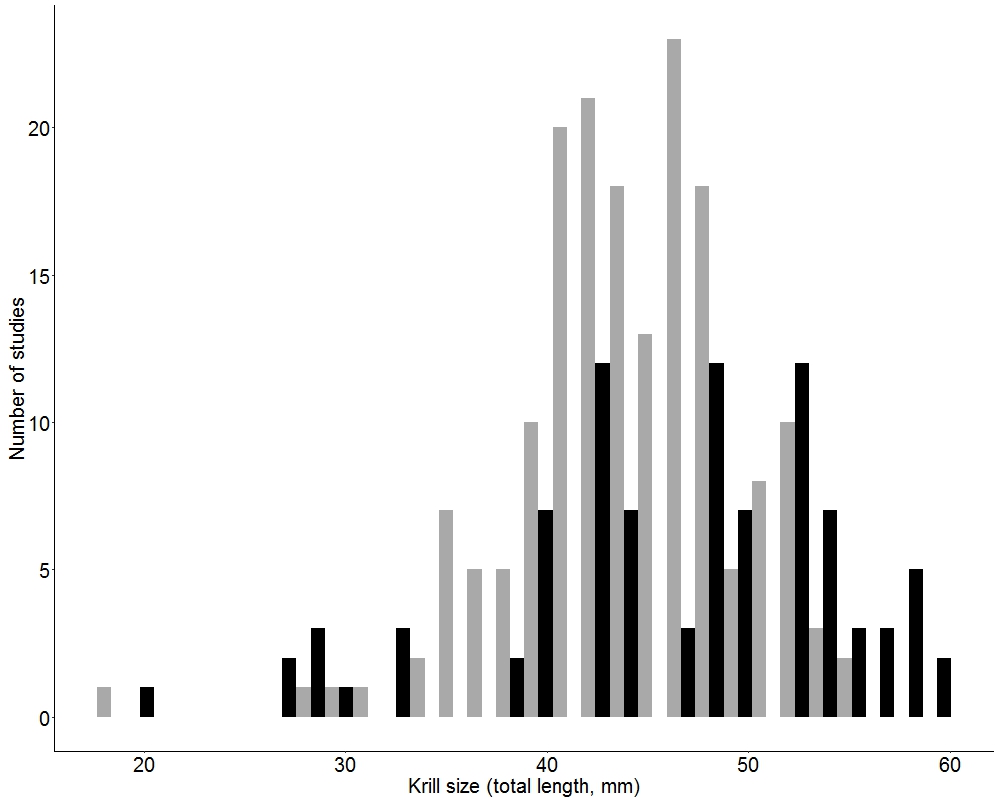


(*b*)


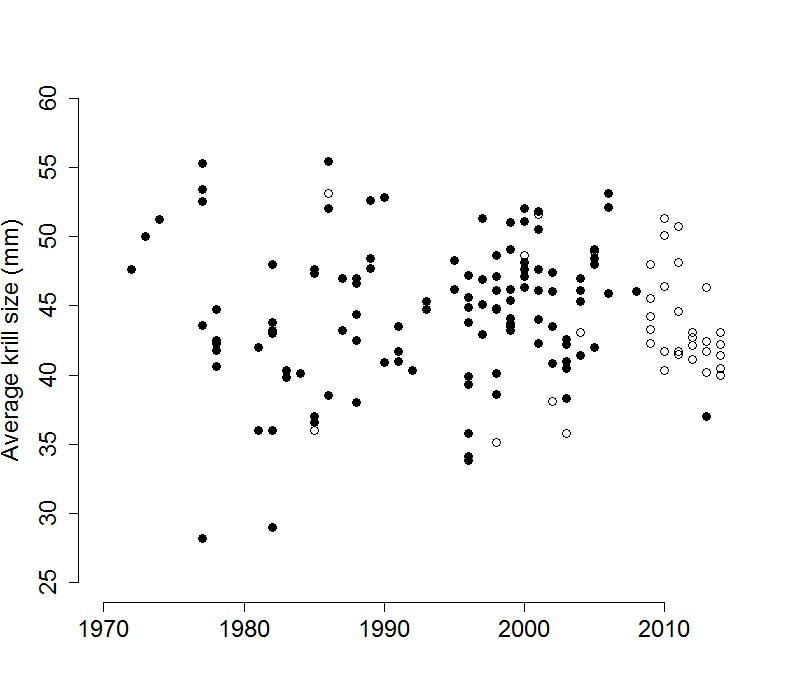

Supplement: S3 Fig — (DOCX) [file pone.0156968.s003.docx]
