# Supplementary material for: At-Sea Distribution and Prey Selection of Antarctic Petrels and Commercial Krill Fisheries
Source: PLoS One. 2016 Aug 17;11(8):e0156968. doi: 10.1371/journal.pone.0156968 (PMC4988635; doi:10.1371/journal.pone.0156968)
Supplement: S1 Table — (DOCX) [file pone.0156968.s004.docx]

***S1 Table.*** *Summary statistics for isotopic ratios of carbon (δ^13^C) and nitrogen (δ^15^N) measured in Antarctic petrel body feathers (N = 133). All samples were pooled over three consecutive years, from 2011-12 to 2013-14. These values reflect the isotopic ratios of the prey items that comprised the diet at the time of feather growth, outside the breeding season [1]. Feather δ^15^N values show large variation and are on average rather low, pointing at the contribution of lower-trophic level prey [likely krill; 2] to the diet of adult Antarctic petrels during their moult. The methods used to obtain the isotopic values for the Antarctic petrel feathers followed exactly those described in Cherel et al. [2].*

| **isotope** | **mean** | **sd** | **min** | **max** |
| --- | --- | --- | --- | --- |
| *δ*^13^C (‰) | -24.9 | 0.7 | -27.0 | -23.0 |
| *δ*^15^N (‰) | 9.1 | 0.8 | 7.6 | 11.5 |

**References**

1. Cherel Y, Quillfeldt P, Delord K, Weimerskirch H. Combination of at-sea activity, geolocation and feather stable isotopes documents where and when seabirds moult. Frontiers in Ecology and Evolution. 2016;4. doi: 10.3389/fevo.2016.00003.

2. Cherel Y, Connan M, Jaeger A, Richard P. Seabird year-round and historical feeding ecology: blood and feather δ^13^C and δ^15^N values document foraging plasticity of small sympatric petrels. Mar Ecol Prog Ser. 2014;505:267-80.
