# Supplementary material for: At-Sea Distribution and Prey Selection of Antarctic Petrels and Commercial Krill Fisheries
Source: PLoS One. 2016 Aug 17;11(8):e0156968. doi: 10.1371/journal.pone.0156968 (PMC4988635; doi:10.1371/journal.pone.0156968)
Supplement: S2 Table — (DOCX) [file pone.0156968.s005.docx]

***S4 Table***. *Summary of the literature review. Mean and modal size (total length, TL) of Antarctic krill harvested by top predators (seabirds and seals) and by scientific or commercial trawls. Most values of the modal size and range are estimated from figures. For the scientific trawls, only the ones performed as part of a study on seabirds or seals have been included. Method “M” refers to direct measurement of intact krill specimens; method “A” refers to the use of allometric equation to estimate krill TL. Mean and modal TL are given in mm. All references are detailed at the end this document.*

| **Predator** | **Place name** | **Lat** | **Long** | **Season** | **YR** | **Mean TL** | **Modal TL** | **Method** | **Reference** |
| --- | --- | --- | --- | --- | --- | --- | --- | --- | --- |
| Adélie penguin | Bechervaise Island, East Antarctica | 67°35'S | 62°49'E | Summer | 2001 |  | 48 | A+M | Nicol et al. 2008 |
| Adélie penguin | Bechervaise Island, East Antarctica | 67°35'S | 62°49'E | Summer | 2003 |  | 44 | A+M | Nicol et al. 2008 |
| Adélie penguin | Esperanza Bay, Antarctic Peninsula | 63°23'S | 57°00'W | Summer | 1988 | 38 | 40 | M | Coria et al. 1995 |
| Adélie penguin | King George Island, South Shetlands | 62°10'S | 58°30'W | Summer | 1978 | 41.8 |  | M | Trivelpiece et al. 1990 |
| Adélie penguin | King George Island, South Shetlands | 62°10'S | 58°30'W | Summer | 1978 | 40.6 |  | M | Volkman et al. 1980 |
| Adélie penguin | King George Island, South Shetlands | 62°10'S | 58°30'W | Summer | 1982 | 43.2 |  | M | Trivelpiece et al. 1990 |
| Adélie penguin | King George Island, South Shetlands | 62°10'S | 58°30'W | Summer | 1983 | 39.8 |  | M | Trivelpiece et al. 1990 |
| Adélie penguin | Laurie Island, South Orkneys | 60°46'S | 44°42'W | Summer | 1998 | 44.7 |  | M | Rombola et al. 2012 |
| Adélie penguin | Laurie Island, South Orkneys | 60°46'S | 44°42'W | Summer | 1999 | 43.2 |  | M | Rombola et al. 2012 |
| Adélie penguin | Laurie Island, South Orkneys | 60°46'S | 44°42'W | Summer | 2000 | 47.1 |  | M | Rombola et al. 2012 |
| Adélie penguin | Laurie Island, South Orkneys | 60°46'S | 44°42'W | Summer | 2001 | 42.3 |  | M | Rombola et al. 2012 |
| Adélie penguin | Laurie Island, South Orkneys | 60°46'S | 44°42'W | Summer | 2002 | 46 |  | M | Rombola et al. 2012 |
| Adélie penguin | Laurie Island, South Orkneys | 60°46'S | 44°42'W | Summer | 2003 | 40.5 |  | M | Rombola et al. 2012 |
| Adélie penguin | Laurie Island, South Orkneys | 60°46'S | 44°42'W | Summer | 2004 | 45.3 |  | M | Rombola et al. 2012 |
| Adélie penguin | Laurie Island, South Orkneys | 60°46'S | 44°42'W | Summer | 2005 | 49.1 |  | M | Rombola et al. 2012 |
| Adélie penguin | Pointe Géologie, Adélie Land | 66°40’S | 140°01'E | Summer | 1982 | 43 |  | A | Ridoux & Offredo 1989 |
| Adélie penguin | Prince Olav Coast, Enderby Land | 68°08'S | 42°39'E | Summer | 1990 | 40.9 |  | A | Watanuki et al. 1994 |
| Adélie penguin | Prince Olav Coast, Enderby Land | 68°08'S | 42°39'E | Summer | 1991 | 41 |  | A | Watanuki et al. 1994 |
| Adélie penguin | Prince Olav Coast, Enderby Land | 68°08'S | 42°39'E | Summer | 1991 | 41.7 |  | A | Watanuki et al. 1994 |
| Adélie penguin | Prydz Bay, East Antarctica | 68°35'S | 77°54'E | Summer | 1984 | 40.1 |  | A | Green & Johnstone 1988 |
| Adélie penguin | Prydz Bay, East Antarctica | 68°35'S | 77°54'E | Summer | 1985 | 36.6 |  | A | Green & Johnstone 1988 |
| Adélie penguin | Prydz Bay, East Antarctica | 68°30'S | 77°50'W | Summer | 1983-1984 | 40.6 |  | A | Puddicombe & Johnstone 1988 |
| Adélie penguin | Ross Sea | 70°00'S | 170°00'E | Summer | 1978-80 | 40 |  | M | Ainley et al. 1984 |
| Adélie penguin | Signy Island, South Orkneys | 60°72'S | 45°36'E | Summer | 1981 | 36 | 40 | M | Lishman 1985 |
| Adélie penguin | Signy Island, South Orkneys | 60°72'S | 45°36'E | Summer | 1982 | 29 | 20 | M | Lishman 1985 |
| Adélie penguin | Signy Island, South Orkneys | 60°43'S | 45°34'E | Summer | 1997 | 42.9 | 42 | A | Lynnes et al. 2004 |
| Adélie penguin | Signy Island, South Orkneys | 60°43'S | 45°34'E | Summer | 1998 | 47.1 | 48 | A | Lynnes et al. 2004 |
| Adélie penguin | Signy Island, South Orkneys | 60°43'S | 45°34'E | Summer | 1999 | 44.1 | 48 | A | Lynnes et al. 2004 |
| Adélie penguin | Signy Island, South Orkneys | 60°43'S | 45°34'E | Summer | 2000 |  | 54 | A | Lynnes et al. 2002 |
| Adélie penguin | Signy Island, South Orkneys | 60°43'S | 45°34'E | Summer | 2000 | 51.1 | 54 | A | Lynnes et al. 2004 |
| Adélie penguin | Signy Island, South Orkneys | 60°43'S | 45°34'E | Summer | 2001 |  | 52 | A | Lynnes et al. 2002 |
| Adélie penguin | Signy Island, South Orkneys | 60°43'S | 45°34'E | Summer | 2001 | 44 | 52 | A | Lynnes et al. 2004 |
| Antarctic fulmar | Pointe Géologie, Adélie Land | 66°40’S | 140°01'E | Summer | 1982 | 43 |  | A | Ridoux & Offredo 1989 |
| Antarctic fur seal | Bird Island, South Georgia | 54°00'S | 38°02'W | Summer | 1972 | 47.6 |  | M | Croxall & Pilcher 1984 |
| Antarctic fur seal | Bird Island, South Georgia | 54°00'S | 38°02'W | Summer | 1973 | 50 |  | M | Croxall & Pilcher 1984 |
| Antarctic fur seal | Bird Island, South Georgia | 54°00'S | 38°02'W | Summer | 1974 | 51.2 |  | M | Croxall & Pilcher 1984 |
| Antarctic fur seal | Bird Island, South Georgia | 54°00'S | 38°02'W | Summer | 1977 | 53.4 |  | M | Croxall & Pilcher 1984 |
| Antarctic fur seal | Bird Island, South Georgia | 54°00'S | 38°02'W | Summer | 1977 | 55.3 |  | M | Croxall & Pilcher 1984 |
| Antarctic fur seal | Bird Island, South Georgia | 54°00'S | 38°02'W | Summer | 1989 | 52.6 |  | A | Boyd et al. 1991 |
| Antarctic fur seal | Bird Island, South Georgia | 54°00'S | 38°02'W | Summer | 1990 | 52.8 |  | A | Boyd et al. 1991 |
| Antarctic fur seal | Bird Island, South Georgia | 54°00'S | 38°02'W | Summer | 1992 | 40.3 | 42 | M | Reid 1995 |
| Antarctic fur seal | Bird Island, South Georgia | 54°00'S | 38°02'W | Winter | 1993 | 44.7 | 44 | M | Reid 1995 |
| Antarctic fur seal | Bird Island, South Georgia | 54°00'S | 38°02'W | Winter | 1996 | 35.8 |  | A | Berrow et al. 1999 |
| Antarctic fur seal | Bird Island, South Georgia | 54°00'S | 38°02'W | Winter | 1996 | 39.3 |  | A | Berrow et al. 1999 |
| Antarctic fur seal | Bird Island, South Georgia | 54°00'S | 38°02'W | Winter | 1996 | 45.6 |  | A | Berrow et al. 1999 |
| Antarctic fur seal | Bird Island, South Georgia | 54°00'S | 38°02'W | Summer | 1997 | 46.9 |  | A | Berrow et al. 1999 |
| Antarctic fur seal | Bird Island, South Georgia | 54°00'S | 38°02'W | Summer | 1999 |  | 56 | A | Barlow et al. 2002 |
| Antarctic fur seal | Bird Island, South Georgia | 54°00'S | 38°02'W | Summer | 2000 |  | 42 | A | Barlow et al. 2002 |
| Antarctic fur seal | Bird Island, South Georgia | 54°00'S | 38°02'W | Summer | 2000 |  | 44 | A | Reid et al. 2004 |
| Antarctic fur seal | Bird Island, South Georgia | 54°00'S | 38°02'W | Summer | 1991-1994 |  | 42 | M | Reid & Arnould 1996 |
| Antarctic fur seal | Bouvet Island | 54°25'S | 03°20'E | Summer | 1999 | 51 | 50 | M | Kirkman et al. 2000 |
| Antarctic fur seal | Livingston Island, South Shetlands | 62°28'S | 60°47'W | Summer | 2000 |  | 52 | A | Reid et al. 2004 |
| Antarctic fur seal | Signy Island, South Orkneys | 60°43'S | 45°34'E | Summer | 2000 |  | 48 | A | Reid et al. 2004 |
| Antarctic fur seal | South Georgia | ? | ? | Summer | 1986 |  | 56 | A | Croxall et al. 1999 |
| Antarctic fur seal | South Georgia | ? | ? | Summer | 1994 |  | 40 | A | Croxall et al. 1999 |
| Antarctic petrel | Prydz Bay, East Antarctica | 67°00'S | 75°00'E | Summer | 1982 | 48 | 48 | M | Montague 1984 |
| Antarctic petrel | Prydz Bay, East Antarctica | 67°47'S | 66°42'E | Summer | 1987 | 47 |  | A | Klages et al. 1990 |
| Antarctic petrel | Prydz Bay, East Antarctica | 68°50'S | 77°50'E | Summer | 1988 | 46.6 |  | A | Arnould & Whitehead 1991 |
| Antarctic petrel | Prydz Bay, East Antarctica | 68°50'S | 77°42'E | Summer | 1989 | 48.4 |  | A | Norman & Ward 1992 |
| Antarctic petrel | Prydz Bay, East Antarctica | 67°00'S | 75°00'E | Summer | 1991 | 43.5 |  | M | Nicol 1993 |
| Antarctic petrel | Ross Sea | 70°00'S | 170°00'E | Summer | 1978-80 | 37 |  | M | Ainley et al. 1984 |
| Antarctic petrel | Svarthamaren, Dronning Maud Land | 71°53'S | 05°10'E | Summer | 2013 | 37 | 29 | A | this study |
| Antarctic petrel | Svarthamaren, Dronning Maud Land | 71°53'S | 05°10'E | Summer | 1992 and 1994 | 44 | 48 | A | Lorentsen et al. 1998 |
| Antarctic prion | Ross Sea | 70°00'S | 170°00'E | Summer | 1978-80 | 19 |  | M | Ainley et al. 1984 |
| Black-browed albatross | Bird Island, South Georgia | 54°00'S | 38°02'W | Summer | 1986 |  | 56 | A | Reid et al. 1996, Croxall et al. 1997 |
| Blue petrel | Mayes Island, Kerguelen | 49°28'S | 69°57'W | Summer | 1995 to 1998 | 46.8 |  | A | Cherel et al. 2002 |
| Cape petrel | ? | ? | ? | ? | ? | 41.7 |  | ? | Arnould & Whitehead 1991 ^a^ |
| Cape petrel | Laurie Island, South Orkneys | 60°46'S | 44°42'W | Winter | 1993 | 45.3 |  | M | Coria et al. 2000 |
| Cape petrel | Laurie Island, South Orkneys | 60°46'S | 44°42'W | Winter | 1995 | 46.2 |  | M | Coria et al. 2000 |
| Cape petrel | Laurie Island, South Orkneys | 60°46'S | 44°42'W | Summer | 1995 | 48.3 |  | A | Soave et al. 1996 |
| Cape petrel | Laurie Island, South Orkneys | 60°46'S | 44°42'W | Summer | 1996 | 39.9 |  | A | Coria et al. 1997 |
| Cape petrel | Laurie Island, South Orkneys | 60°46'S | 44°42'W | Winter | 1996 | 44.9 |  | M | Coria et al. 2000 |
| Cape petrel | Pointe Géologie, Adélie Land | 66°40’S | 140°01'E | Summer | 1982 | 43 |  | A | Ridoux & Offredo 1989 |
| Cape petrel | Prydz Bay, East Antarctica | 68°50'S | 77°50'E | Summer | 1988 | 46.6 |  | A | Arnould & Whitehead 1991 |
| Cape petrel | Ross Sea | 70°00'S | 170°00'E | Summer | 1978-80 | 44 |  | M | Ainley et al. 1984 |
| Chinstrap penguin | Elephant Island, South Shetlands | 61°00'S | 55°00'W | Summer | 1977 | 43.6 |  | M | Croxall & Furse 1980 |
| Chinstrap penguin | Elephant Island, South Shetlands | 61°00'S | 55°00'W | Summer | 1988 |  | 44 | A | Ichii et al. 1996 |
| Chinstrap penguin | King George Island, South Shetlands | 62°10'S | 58°30'W | Summer | 1978 | 42.5 |  | M | Trivelpiece et al. 1990 |
| Chinstrap penguin | King George Island, South Shetlands | 62°10'S | 58°30'W | Summer | 1978 | 42.3 |  | M | Volkman et al. 1980 |
| Chinstrap penguin | King George Island, South Shetlands | 62°10'S | 58°30'W | Summer | 1982 | 43.8 |  | M | Trivelpiece et al. 1990 |
| Chinstrap penguin | King George Island, South Shetlands | 62°10'S | 58°30'W | Summer | 1983 | 40.3 |  | M | Trivelpiece et al. 1990 |
| Chinstrap penguin | Laurie Island, South Orkneys | 60°46'S | 44°42'W | Summer | 1998 | 44.8 |  | M | Rombola et al. 2012 |
| Chinstrap penguin | Laurie Island, South Orkneys | 60°46'S | 44°42'W | Summer | 1999 | 46.2 |  | M | Rombola et al. 2012 |
| Chinstrap penguin | Laurie Island, South Orkneys | 60°46'S | 44°42'W | Summer | 2000 | 46.3 |  | M | Rombola et al. 2012 |
| Chinstrap penguin | Laurie Island, South Orkneys | 60°46'S | 44°42'W | Summer | 2001 | 46.1 |  | M | Rombola et al. 2012 |
| Chinstrap penguin | Laurie Island, South Orkneys | 60°46'S | 44°42'W | Summer | 2002 | 40.8 |  | M | Rombola et al. 2012 |
| Chinstrap penguin | Laurie Island, South Orkneys | 60°46'S | 44°42'W | Summer | 2003 | 42.6 |  | M | Rombola et al. 2012 |
| Chinstrap penguin | Laurie Island, South Orkneys | 60°46'S | 44°42'W | Summer | 2004 | 46.1 |  | M | Rombola et al. 2012 |
| Chinstrap penguin | Laurie Island, South Orkneys | 60°46'S | 44°42'W | Summer | 2005 | 48.4 |  | M | Rombola et al. 2012 |
| Chinstrap penguin | Laurie Island, South Orkneys | 60°46'S | 44°42'W | Summer | 2006 | 45.9 |  | M | Rombola et al. 2012 |
| Chinstrap penguin | Livingston Island, South Shetlands | 62°28'S | 60°47'W | Summer | 1998 | 38.6 |  | M | Miller & Trivelpiece 2007 |
| Chinstrap penguin | Livingston Island, South Shetlands | 62°28'S | 60°47'W | Summer | 1999 | 43.7 |  | M | Miller & Trivelpiece 2007 |
| Chinstrap penguin | Livingston Island, South Shetlands | 62°28'S | 60°47'W | Summer | 2000 | 47.6 |  | M | Miller & Trivelpiece 2007 |
| Chinstrap penguin | Livingston Island, South Shetlands | 62°28'S | 60°47'W | Summer | 2001 | 50.5 |  | M | Miller & Trivelpiece 2007 |
| Chinstrap penguin | Livingston Island, South Shetlands | 62°28'S | 60°47'W | Summer | 2002 | 43.5 |  | M | Miller & Trivelpiece 2007 |
| Chinstrap penguin | Livingston Island, South Shetlands | 62°28'S | 60°47'W | Summer | 2003 | 38.3 |  | M | Miller & Trivelpiece 2007 |
| Chinstrap penguin | Livingston Island, South Shetlands | 62°28'S | 60°47'W | Summer | 2004 | 41.4 |  | M | Miller & Trivelpiece 2007 |
| Chinstrap penguin | Livingston Island, South Shetlands | 62°28'S | 60°47'W | Summer | 2005 | 48 |  | M | Miller & Trivelpiece 2007 |
| Chinstrap penguin | Livingston Island, South Shetlands | 62°28'S | 60°47'W | Summer | 2006 | 52.1 |  | M | Miller & Trivelpiece 2007 |
| Chinstrap penguin | Signy Island, South Orkneys | 60°72'S | 45°36'E | Summer | 1981 | 42 | 40 | M | Lishman 1985 |
| Chinstrap penguin | Signy Island, South Orkneys | 60°72'S | 45°36'E | Summer | 1982 | 36 | 40 | M | Lishman 1985 |
| Chinstrap penguin | Signy Island, South Orkneys | 60°43'S | 45°34'E | Summer | 1997 | 45.1 | 46 | A | Lynnes et al. 2004 |
| Chinstrap penguin | Signy Island, South Orkneys | 60°43'S | 45°34'E | Summer | 1998 | 48.6 | 50 | A | Lynnes et al. 2004 |
| Chinstrap penguin | Signy Island, South Orkneys | 60°43'S | 45°34'E | Summer | 1999 | 49.1 | 52 | A | Lynnes et al. 2004 |
| Chinstrap penguin | Signy Island, South Orkneys | 60°43'S | 45°34'E | Summer | 2000 |  | 54 | A | Lynnes et al. 2002 |
| Chinstrap penguin | Signy Island, South Orkneys | 60°43'S | 45°34'E | Summer | 2000 | 52 | 54 | A | Lynnes et al. 2004 |
| Chinstrap penguin | Signy Island, South Orkneys | 60°43'S | 45°34'E | Summer | 2001 |  | 52 | A | Lynnes et al. 2002 |
| Chinstrap penguin | Signy Island, South Orkneys | 60°43'S | 45°34'E | Summer | 2001 | 47.6 | 52 | A | Lynnes et al. 2004 |
| Chinstrap penguin | Signy Island, South Orkneys | 60°72'S | 45°36'E | Summer | ? | 31.6 |  | ? | Croxall & Furse 1980 ^b^ |
| Commercial trawls | Elephant Island, South Shetlands | 61°00'S | 55°00'W | Summer | 1988 |  | 44 | M | Ichii et al. 1996 |
| Commercial trawls | South Shetlands | 60-63°00'S | 53-63°00'W | Summer | 2000 | 48.6 | 50 | M | Jones & Ramm 2004 |
| Commercial trawls | subarea 48.1 |  |  | Summer | 2009 | 43.3 |  | M | CCAMLR |
| Commercial trawls | subarea 48.1 |  |  | Winter | 2009 | 44.2 |  | M | CCAMLR |
| Commercial trawls | subarea 48.1 |  |  | Winter | 2010 | 40.3 |  | M | CCAMLR |
| Commercial trawls | subarea 48.1 |  |  | Summer | 2010 | 51.3 |  | M | CCAMLR |
| Commercial trawls | subarea 48.1 |  |  | Winter | 2011 | 48.1 |  | M | CCAMLR |
| Commercial trawls | subarea 48.1 |  |  | Summer | 2011 | 50.7 |  | M | CCAMLR |
| Commercial trawls | subarea 48.1 |  |  | Summer | 2012 | 42.7 |  | M | CCAMLR |
| Commercial trawls | subarea 48.1 |  |  | Winter | 2012 | 43.1 |  | M | CCAMLR |
| Commercial trawls | subarea 48.1 |  |  | Winter | 2013 | 40.2 |  | M | CCAMLR |
| Commercial trawls | subarea 48.1 |  |  | Summer | 2013 | 41.7 |  | M | CCAMLR |
| Commercial trawls | subarea 48.1 |  |  | Winter | 2014 | 40 |  | M | CCAMLR |
| Commercial trawls | subarea 48.1 |  |  | Summer | 2014 | 42.2 |  | M | CCAMLR |
| Commercial trawls | subarea 48.2 |  |  | Summer | 2009 | 42.3 |  | M | CCAMLR |
| Commercial trawls | subarea 48.2 |  |  | Winter | 2009 | 45.5 |  | M | CCAMLR |
| Commercial trawls | subarea 48.2 |  |  | Winter | 2010 | 46.4 |  | M | CCAMLR |
| Commercial trawls | subarea 48.2 |  |  | Summer | 2010 | 50.1 |  | M | CCAMLR |
| Commercial trawls | subarea 48.2 |  |  | Summer | 2011 | 41.5 |  | M | CCAMLR |
| Commercial trawls | subarea 48.2 |  |  | Winter | 2011 | 44.6 |  | M | CCAMLR |
| Commercial trawls | subarea 48.2 |  |  | Summer | 2012 | 41.1 |  | M | CCAMLR |
| Commercial trawls | subarea 48.2 |  |  | Winter | 2013 | 42.4 |  | M | CCAMLR |
| Commercial trawls | subarea 48.2 |  |  | Winter | 2014 | 40.5 |  | M | CCAMLR |
| Commercial trawls | subarea 48.2 |  |  | Summer | 2014 | 43.1 |  | M | CCAMLR |
| Commercial trawls | subarea 48.3 |  |  | Winter | 2009 | 48 |  | M | CCAMLR |
| Commercial trawls | subarea 48.3 |  |  | Winter | 2010 | 41.7 |  | M | CCAMLR |
| Commercial trawls | subarea 48.3 |  |  | Winter | 2011 | 41.7 |  | M | CCAMLR |
| Commercial trawls | subarea 48.3 |  |  | Winter | 2012 | 42.1 |  | M | CCAMLR |
| Commercial trawls | subarea 48.3 |  |  | Winter | 2013 | 46.3 |  | M | CCAMLR |
| Commercial trawls | subarea 48.3 |  |  | Winter | 2014 | 41.4 |  | M | CCAMLR |
| Crabeater seal | Bismarck Strait, Antarctic Peninsula | 65°00'S | 65°00'W | Winter | 1985 | 47.6 |  | M | Lowry et al. 1988 |
| Emperor penguin | Dresher Inlet, Weddell Sea | 72°52'S | 19°25'W | Winter | 1986 | 38.5 |  | M | Klages 1989 |
| Gentoo penguin | Bird Island, South Georgia | 54°00'S | 38°02'W | Summer | 1985 | 37 |  | M | Croxall et al. 1988 |
| Gentoo penguin | Bird Island, South Georgia | 54°00'S | 38°02'W | Summer | 1986 |  | 58 | A | Reid et al. 1996, Croxall et al. 1997 |
| Gentoo penguin | Bird Island, South Georgia | 54°00'S | 38°02'W | Summer | 1986 | 52 |  | M | Croxall et al. 1988 |
| Gentoo penguin | Bird Island, South Georgia | 54°00'S | 38°02'W | Winter | 1988 | 42.5 |  | A | Williams 1991 |
| Gentoo penguin | Bird Island, South Georgia | 54°00'S | 38°02'W | Winter | 1996 | 33.8 |  | A | Berrow et al. 1999 |
| Gentoo penguin | Bird Island, South Georgia | 54°00'S | 38°02'W | Winter | 1996 | 34.1 |  | A | Berrow et al. 1999 |
| Gentoo penguin | Bird Island, South Georgia | 54°00'S | 38°02'W | Winter | 1996 | 43.8 |  | A | Berrow et al. 1999 |
| Gentoo penguin | Bird Island, South Georgia | 54°00'S | 38°02'W | Summer | 1997 | 51.3 |  | A | Berrow et al. 1999 |
| Gentoo penguin | King George Island, South Shetlands | 62°10'S | 58°30'W | Summer | 1978 | 44.7 |  | M | Volkman et al. 1980 |
| Gentoo penguin | Livingston Island, South Shetlands | 62°28'S | 60°47'W | Summer | 1998 | 40.1 |  | M | Miller & Trivelpiece 2007 |
| Gentoo penguin | Livingston Island, South Shetlands | 62°28'S | 60°47'W | Summer | 1999 | 45.4 |  | M | Miller & Trivelpiece 2007 |
| Gentoo penguin | Livingston Island, South Shetlands | 62°28'S | 60°47'W | Summer | 2000 | 48.1 |  | M | Miller & Trivelpiece 2007 |
| Gentoo penguin | Livingston Island, South Shetlands | 62°28'S | 60°47'W | Summer | 2001 | 51.8 |  | M | Miller & Trivelpiece 2007 |
| Gentoo penguin | Livingston Island, South Shetlands | 62°28'S | 60°47'W | Summer | 2002 | 46 |  | M | Miller et al. 2009 |
| Gentoo penguin | Livingston Island, South Shetlands | 62°28'S | 60°47'W | Summer | 2002 | 47.4 |  | M | Miller & Trivelpiece 2007 |
| Gentoo penguin | Livingston Island, South Shetlands | 62°28'S | 60°47'W | Summer | 2003 | 41 |  | M | Miller et al. 2009 |
| Gentoo penguin | Livingston Island, South Shetlands | 62°28'S | 60°47'W | Summer | 2003 | 42.2 |  | M | Miller & Trivelpiece 2007 |
| Gentoo penguin | Livingston Island, South Shetlands | 62°28'S | 60°47'W | Summer | 2004 | 47 |  | M | Miller et al. 2009 |
| Gentoo penguin | Livingston Island, South Shetlands | 62°28'S | 60°47'W | Summer | 2004 | 47 |  | M | Miller & Trivelpiece 2007 |
| Gentoo penguin | Livingston Island, South Shetlands | 62°28'S | 60°47'W | Summer | 2005 | 42 |  | M | Miller et al. 2009 |
| Gentoo penguin | Livingston Island, South Shetlands | 62°28'S | 60°47'W | Summer | 2005 | 48.4 |  | M | Miller & Trivelpiece 2007 |
| Gentoo penguin | Livingston Island, South Shetlands | 62°28'S | 60°47'W | Summer | 2006 | 53.1 |  | M | Miller & Trivelpiece 2007 |
| Gentoo penguin | Livingston Island, South Shetlands | 62°28'S | 60°47'W | Summer | 2008 | 46 |  | M | Miller et al. 2009 |
| Gentoo penguin | South Georgia | ? | ? | Summer | 1986 |  | 58 | A | Croxall et al. 1999 |
| Gentoo penguin | South Georgia | ? | ? | Summer | 1994 |  | 38 | A | Croxall et al. 1999 |
| Gentoo penguin | Square Pond, Bird Island, South Georgia | 54°00'S | 38°02'W | Winter | 1987 | 43.2 |  | A | Williams 1991 |
| Gentoo penguin | Square Pond, Bird Island, South Georgia | 54°00'S | 38°02'W | Winter | 1988 | 44.4 |  | A | Williams 1991 |
| Gentoo penguin, Macaroni penguin, Antarctic fur seal | Bird Island, South Georgia | 54°00'S | 38°02'W | Summer | 1992 |  | 42 | A | Reid et al. 1999 |
| Gentoo penguin, Macaroni penguin, Antarctic fur seal | Bird Island, South Georgia | 54°00'S | 38°02'W | Summer | 1993 |  | 46 | A | Reid et al. 1999 |
| Gentoo penguin, Macaroni penguin, Antarctic fur seal | Bird Island, South Georgia | 54°00'S | 38°02'W | Summer | 1994 |  | 42 | A | Reid et al. 1999 |
| Gentoo penguin, Macaroni penguin, Antarctic fur seal | Bird Island, South Georgia | 54°00'S | 38°02'W | Summer | 1995 |  | 50 | A | Reid et al. 1999 |
| Gentoo penguin, Macaroni penguin, Antarctic fur seal | Bird Island, South Georgia | 54°00'S | 38°02'W | Summer | 1996 |  | 42 | A | Reid et al. 1999 |
| Gentoo penguin, Macaroni penguin, Antarctic fur seal | Bird Island, South Georgia | 54°00'S | 38°02'W | Summer | 1997 |  | 44 | A | Reid et al. 1999 |
| Grey-headed albatross | Bird Island, South Georgia | 54°00'S | 38°02'W | Summer | 1986 |  | 52 | A | Reid et al. 1996, Croxall et al. 1997 |
| Grey-headed albatross | Bird Island, South Georgia | 54°00'S | 38°02'W | Summer | 2000 |  | 50 | M | Xavier et al. 2003 |
| Grey-headed and black-browed albatross | Bird Island, South Georgia | 54°00'S | 36°00'W | Summer | 1975, 1976 | 53 |  | M | Prince 1980 |
| Leopard seal | Bismarck Strait, Antarctic Peninsula | 65°00'S | 65°00'W | Winter | 1985 | 47.3 |  | M | Lowry et al. 1988 |
| Light-Mantled Sooty Albatross | Bird Island, South Georgia | 54°00'S | 38°02'W | Summer | 1977 | 52.5 |  | M | Thomas 1982 |
| Light-Mantled Sooty Albatross | Ross Sea | 70°00'S | 170°00'E | Summer | 1978-80 | 39 |  | M | Ainley et al. 1984 |
| Macaroni penguin | Bird Island, South Georgia | 54°00'S | 38°02'W | Summer | 1986 |  | 58 | A | Reid et al. 1996, Croxall et al. 1997 |
| Macaroni penguin | Bird Island, South Georgia | 54°00'S | 38°02'W | Summer | 1986 | 52 |  | M | Croxall et al. 1988 |
| Macaroni penguin | Bird Island, South Georgia | 54°00'S | 38°02'W | Summer | 1986 | 55.4 | 59 | A | Hill et al. 1996 |
| Macaroni penguin | Bird Island, South Georgia | 54°00'S | 38°02'W | Summer | 1999 |  | 58 | A | Barlow et al. 2002 |
| Macaroni penguin | Bird Island, South Georgia | 54°00'S | 38°02'W | Summer | 2000 |  | 48 | A | Barlow et al. 2002 |
| Macaroni penguin | Elephant Island, South Shetlands | 61°00'S | 55°00'W | Summer | 1977 | 28.2 |  | M | Croxall & Furse 1980 |
| Macaroni penguin | South Georgia | ? | ? | Summer | 1986 |  | 58 | A | Croxall et al. 1999 |
| Macaroni penguin | South Georgia | ? | ? | Summer | 1994 |  | 42 | A | Croxall et al. 1999 |
| Minke whale | Vincennes Bay, East Antarctica | 63°00'S | 110°00'W | Summer | 1988 |  | 27 | M | Ichii et al. 1991 |
| Minke whale | Vincennes Bay, East Antarctica | 65°00'S | 111°00'W | Summer | 1988 |  | 27 | M | Ichii et al. 1991 |
| Minke whale | Vincennes Bay, East Antarctica | 65°00'S | 114°00'W | Summer | 1988 |  | 48 | M | Ichii et al. 1991 |
| Minke whale | Vincennes Bay, East Antarctica | 63°00'S | 114°00'W | Summer | 1988 |  | 46 | M | Ichii et al. 1991 |
| Minke whale | Vincennes Bay, East Antarctica | 61°00'S | 109°00'W | Summer | 1988 |  | 28 | M | Ichii et al. 1991 |
| Minke whale | Vincennes Bay, East Antarctica | 65°00'S | 108°00'W | Summer | 1988 |  | 28 | M | Ichii et al. 1991 |
| Mottled petrel | Ross Sea | 70°00'S | 170°00'E | Summer | 1978-80 | 44 |  | M | Ainley et al. 1984 |
| Scientific trawls | Bechervaise Island, East Antarctica | 67°35'S | 62°49'E | Summer | 2001 |  | 49 | A+M | Nicol et al. 2008 |
| Scientific trawls | Bechervaise Island, East Antarctica | 67°35'S | 62°49'E | Summer | 2003 |  | 49 | A+M | Nicol et al. 2008 |
| Scientific trawls | Bird Island, South Georgia | 54°00'S | 38°02'W | Summer | 1986 |  | 54 | A | Reid et al. 1996, Croxall et al. 1997 |
| Scientific trawls | Bird Island, South Georgia | 54°00'S | 38°02'W | Summer | 1986 |  | 55 | M | Croxall et al. 1995 |
| Scientific trawls | Bird Island, South Georgia | 54°00'S | 38°02'W | Summer | 1986 | 53.1 | 55 | A | Hill et al. 1996 |
| Scientific trawls | Bird Island, South Georgia | 54°00'S | 38°02'W | Summer | 1991 |  | 40 | A | Reid et al. 1999 |
| Scientific trawls | Bird Island, South Georgia | 54°00'S | 38°02'W | Summer | 1993 |  | 44 | A | Reid et al. 1999 |
| Scientific trawls | Bird Island, South Georgia | 54°00'S | 38°02'W | Summer | 1994 |  | 54 | A | Reid et al. 1999 |
| Scientific trawls | Bird Island, South Georgia | 54°00'S | 38°02'W | Summer | 1996 |  | 28 | A | Reid et al. 1999 |
| Scientific trawls | Bird Island, South Georgia | 54°00'S | 38°02'W | Summer | 1997 |  | 38 | A | Reid et al. 1999 |
| Scientific trawls | Bird Island, South Georgia | 54°00'S | 38°02'W | Summer | 2000 |  | 40 | A | Reid et al. 2004 |
| Scientific trawls | Bismarck Strait, Antarctic Peninsula | 65°00'S | 65°00'W | Winter | 1985 | 36 |  | M | Lowry et al. 1988 |
| Scientific trawls | Livingston Island, South Shetlands | 62°28'S | 60°47'W | Summer | 1998 | 35.1 |  | M | Miller & Trivelpiece 2007 |
| Scientific trawls | Livingston Island, South Shetlands | 62°28'S | 60°47'W | Summer | 1999 | 43.5 |  | M | Miller & Trivelpiece 2007 |
| Scientific trawls | Livingston Island, South Shetlands | 62°28'S | 60°47'W | Summer | 2000 | 48.6 |  | M | Miller & Trivelpiece 2007 |
| Scientific trawls | Livingston Island, South Shetlands | 62°28'S | 60°47'W | Summer | 2000 |  | 52 | A | Reid et al. 2004 |
| Scientific trawls | Livingston Island, South Shetlands | 62°28'S | 60°47'W | Summer | 2001 | 51.6 |  | M | Miller & Trivelpiece 2007 |
| Scientific trawls | Livingston Island, South Shetlands | 62°28'S | 60°47'W | Summer | 2002 | 38.1 |  | M | Miller & Trivelpiece 2007 |
| Scientific trawls | Livingston Island, South Shetlands | 62°28'S | 60°47'W | Summer | 2003 | 35.8 |  | M | Miller & Trivelpiece 2007 |
| Scientific trawls | Livingston Island, South Shetlands | 62°28'S | 60°47'W | Summer | 2004 | 43.1 |  | M | Miller & Trivelpiece 2007 |
| Scientific trawls | Livingston Island, South Shetlands | 62°28'S | 60°47'W | Summer | 2005 | 48.9 |  | M | Miller & Trivelpiece 2007 |
| Scientific trawls | Livingston Island, South Shetlands | 62°28'S | 60°47'W | Summer | 2006 | 52.1 |  | M | Miller & Trivelpiece 2007 |
| Scientific trawls | Signy Island, South Orkneys | 60°43'S | 45°34'E | Summer | 2000 |  | 52 | A | Reid et al. 2004 |
| Snow petrel | Ross Sea | 70°00'S | 170°00'E | Summer | 1978-80 | 37 |  | M | Ainley et al. 1984 |
| Southern fulmar | Prydz Bay, East Antarctica | 68°50'S | 77°50'E | Summer | 1988 | 47 |  | A | Arnould & Whitehead 1991 |
| Southern fulmar | Prydz Bay, East Antarctica | 68°50'S | 77°42'E | Summer | 1989 | 47.7 |  | A | Norman & Ward 1992 |
| Southern fulmar | Ross Sea | 70°00'S | 170°00'E | Summer | 1978-80 | 40 |  | M | Ainley et al. 1984 |
| Wandering albatross | Bird Island, South Georgia | 54°00'S | 38°02'W | Summer | 2000 |  | 48 | M | Xavier et al. 2003 |
| White-chinned petrel | Bird Island, South Georgia | 54°00'S | 38°02'W | Summer | 1986 |  | 54 | A | Reid et al. 1996, Croxall et al. 1997 |
| White-chinned petrel | Bird Island, South Georgia | 54°00'S | 38°02'W | Summer | 1986 |  | 55 | M | Croxall et al. 1995 |
| White-chinned petrel | Bird Island, South Georgia | 54°00'S | 38°02'W | Summer | 1996 | 47.2 |  | A | Berrow & Croxall 1999 |
| White-chinned petrel | Bird Island, South Georgia | 54°00'S | 38°02'W | Summer | 1998 | 46.1 | 42 | A | Berrow & Croxall 1999 |
| Wilson's storm petrel | Ross Sea | 70°00'S | 170°00'E | Summer | 1978-80 | 35 |  | M | Ainley et al. 1984 |

^a^: original result from Green 1986

^b^: original result from White & Conroy 1975

**List of references**

Ainley DG, O'Connor EF, Boekelheide RJ 1984 The marine ecology of birds in the Ross Sea, Antarctica, Vol 32. The American Ornithologist Union, Washington, D.C.

Arnould JPY, Whitehead MD 1991 The diet of antarctic petrels, cape petrels and southern fulmars rearing chicks in Prydz Bay. Antarctic Science 3:19-27

Barlow KE, Boyd IL, Croxall JP, Reid K, Staniland IJ, Brierley AS 2002 Are penguins and seals in competition for Antarctic krill at South Georgia? Marine Biology 140:205-213

Berrow SD, Croxall JP 1999 The diet of white-chinned petrels *Procellaria aequinoctialis*, Linnaeus 1758, in years of contrasting prey availability at South Georgia. Antarctic Science 11:283-292

Berrow SD, Taylor RI, Murray AWA 1999 Influence of sampling protocol on diet determination of gentoo penguins *Pygoscelis papua* and Antarctic fur seals Arctocephalus gazella. Polar Biol 22:156-163

Boyd IL, Lunn NJ, Barton T 1991 Time-budgets and foraging characteristics of lactating Antarctic fur seals. J Anim Ecol 60:577-592

Cherel Y, Bocher P, Trouve C, Weimerskirch H 2002 Diet and feeding ecology of blue petrels *Halobaena caerulea* at Iles Kerguelen, Southern Indian Ocean. Mar Ecol Prog Ser 228:283-299

Coria N, Libertelli M, Casaux R, Darrieu C 2000 Inter-annual variation in the autumn diet of the Gentoo Penguin at Laurie Island, Antarctica. Waterbirds 23:511-517

Coria NR, Soave GE, Montalti D 1997 Diet of Cape petrel Daption capense during the post-hatching period at Laurie Island, South Orkney Islands, Antarctica. Polar Biol 18:236-239

Coria NR, Spairani H, Vivequin S, Fontana R 1995 Diet of Adelie penguins *Pygoscelis adeliae* during the post-hatching period at Esperanza Bay, Antarctica, 1987/88. Polar Biol 15:415-418

Croxall JP, Davis RW, Oconnell MJ 1988 Diving patterns in relation to diet of gentoo and macaroni penguins at South Georgia. Condor 90:157-167

Croxall JP, Furse JR 1980 Food of chinstrap penguins *Pygoscelis antarctica* and macaroni penguins *Eudyptes chrysolophus* at Elephant Island group, South Shetland Islands. Ibis 122:237-245

Croxall JP, Hall AJ, Hill HJ, North AW, Rodhouse PG 1995 The food and feeding ecology of the white-chinned petrel *Procellaria aequinoctialis* at South Georgia. J Zool 237:133-150

Croxall JP, Pilcher MN 1984 Characteristics of krill *Euphausia superba* eaten by Antarctic fur seals *Arctocephalus gazella* at South Georgia. British Antarctic Survey Bulletin:117-125

Croxall JP, Prince PA, Reid K 1997 Dietary segregation of krill-eating south Georgia seabirds. J Zool 242:531-556

Croxall JP, Reid K, Prince PA 1999 Diet, provisioning and productivity responses of marine predators to differences in availability of Antarctic krill. Mar Ecol Prog Ser 177:115-131

Green K 1986 Food of the cape pigeon Daption capenre from Princess Elizabeth Land, East Antarctica. Notornis 33:151-154

Green K, Johnstone GW 1988 Changes in the diet of Adelie penguins breeding in East Antarctica. Australian Wildlife Research 15:103-110

Hill HJ, Trathan PN, Croxall JP, Watkins JL 1996 A comparison of Antarctic krill *Euphausia superba* caught by nets and taken by macaroni penguins *Eudyptes chrysolophus*: Evidence for selection? Mar Ecol Prog Ser 140:1-11

Ichii T, Kato H 1991 Food and daily food consumption of southern minke whales in the Antarctic. Polar Biol 11:479-487

Ichii T, Naganobu M, Ogishima T 1996 Competition between the krill fishery and penguins in the South Shetland Islands. Polar Biol 16:63-70

Jones CD, Ramm DC 2004 The commercial harvest of krill in the southwest Atlantic before and during the CCAMLR 2000 Survey. Deep-Sea Res Part II 51:1421-1434

Kirkman SP, Wilson W, Klages NTW, Bester MN, Isaksen K 2000 Diet and estimated food consumption of Antarctic fur seals at Bouvetoya during summer. Polar Biol 23:745-752

Klages N 1989 Food and feeding ecology of Emperor penguins in the Eastern Weddell Sea. Polar Biol 9:385-390

Klages N, Gales R, Pemberton D 1990 The stomach content of Antarctic petrels *Thalassoica antarctica* feeding young chicks at Scullin Monolith, Mawson Coast, Antarctica. Polar Biol 10:545-547

Lishman GS 1985 T The food and feeding ecology of Adelie penguins *Pygoscelis adeliae* and Chinstrap penguins *Pygoscelis antarctica* at Signy Island, South Orkney Islands. J Zool 205:245-263

Lorentsen SH, Klages N, Rov N 1998 Diet and prey consumption of Antarctic petrels *Thalassoica antarctica* at Svarthamaren, Dronning Maud Land, and at sea outside the colony. Polar Biol 19:414-420

Lowry LF, Testa JW, Calvert W 1988 Notes on winter feeding of crab-eater and leopard seals near the Antarctic Peninsula. Polar Biol 8:475-478

Lynnes AS, Reid K, Croxall JP 2004 Diet and reproductive success of Adelie and chinstrap penguins: linking response of predators to prey population dynamics. Polar Biol 27:544-554

Lynnes AS, Reid K, Croxall JP, Trathan PN 2002 Conflict or co-existence? Foraging distribution and competition for prey between Adelie and chinstrap penguins. Marine Biology 141:1165-1174

Miller AK, Karnovsky NJ, Trivelpiece WZ 2009 Flexible foraging strategies of gentoo penguins *Pygoscelis papua* over 5 years in the South Shetland Islands, Antarctica. Marine Biology 156:2527-2537

Miller AK, Trivelpiece WZ 2007 Cycles of *Euphausia superba* recruitment evident in the diet of Pygoscelid penguins and net trawls in the South Shetland Islands, Antarctica. Polar Biol 30:1615-1623

Montague TL 1984 The food of Antarctic petrels *Thalassoica antarctica*. Emu 84:244-245

Nicol S 1993 A comparison of Antarctic petrel Thalassoica antarctica diets with net samples of Antarctic krill *Euphausia superba* taken from the Prydz Bay region. Polar Biol 13:399-403

Nicol S, Clarke J, Romaine SJ, Kawaguchi S, Williams G, Hosie GW 2008 Krill *Euphausia superba* abundance and Adelie penguin *Pygoscelis adeliae* breeding performance in the waters off the Bechervaise Island colony, East Antarctica in 2 years with contrasting ecological conditions. Deep-Sea Res Part II 55:540-557

Norman FI, Ward SJ 1992 Food and aspects of growth in the Antarctic petrel and southern fulmar breeding at Hop Island, Rauer group, East Antarctica. Emu 92:207-222

Prince PA 1980 The food and feeding ecology of Grey-headed albatross *Diomedea chrysostoma* and black-browed albatross *Diomedea melanophris*. Ibis 122:476-488

Puddicombe RA, Johnstone GW 1988 The breeding-season diet of Adelie penguins at the Vestfold Hills, East Antarctica. Hydrobiologia 165:239-253

Reid K 1995 The diet of Antarctic fur seals *Arctocephallus gazella* Peters 1875 during winter at South Georgia. Antarctic Science 7:241-249

Reid K, Arnould JPY 1996 The diet of Antarctic fur seals *Arctocephalus gazella* during the breeding season at South Georgia. Polar Biol 16:105-114

Reid K, Sims M, White RW, Gillon KW 2004 Spatial distribution of predator/prey interactions in the Scotia Sea: implications for measuring predator/fisheries overlap. Deep-Sea Res Part II 51:1383-1396

Reid K, Trathan PN, Croxall JP, Hill HJ 1996 Krill caught by predators and nets: Differences between species and techniques. Mar Ecol Prog Ser 140:13-20

Reid K, Watkins JL, Croxall JP, Murphy EJ 1999 Krill population dynamics at South Georgia 1991-1997, based on data from predators and nets. Mar Ecol Prog Ser 177:103-114

Ridoux V, Offredo C 1989 The diet of 5 summer breeding seabirds in Adelie Land, Antarctica. Polar Biol 9:137-145

Rombola E, Marschoff E, Coria N 2012 Analysis of the sources of variance in the mean size of krill consumed by Chinstrap and Adelie penguins at South Orkney Islands. Polar Biol 35:1601-1606

Soave GE, Coria NR, Montalti D 1996 Diet of the pintado petrel *Daption capense* during the late incubation and chick-rearing periods at Laurie Island, South Orkney Islands, Antarctica, January-February 1995. Marine Ornithology 24:35-37

Thomas G 1982 The food and feeding ecology of the light-mantled sooty albatross at South Georgia. Emu 82:92-100

Trivelpiece WZ, Trivelpiece SG, Geupel GR, Kjelmyr J, Volkman NJ 1990 Adélie and Chinstrap Penguins: Their Potential as Monitors of the Southern Ocean Marine Ecosystem. In: Kerry KR, Hempel G eds Antarctic Ecosystems, Ecological Change and Conservation. Springer-Verlag, Berlin Heidelberg

Volkman NJ, Presler P, Trivelpiece W 1980 Diet of Pygoscelid penguins at King George Island, Antarctica. Condor 82:373-378

Watanuki Y, Mori Y, Naito Y 1994 *Euphausia superba* dominates in the diet of Adelie penguins feeding under fast sea-ice in the shelf areas of Enderby Land in summer. Polar Biol 14:429-432

White MG, Conroy JWH 1975 Aspects of competition between pygoscelid penguins at Signy Island, South Orkney Islands. Ibis 118:371-373

Williams TD 1991 Foraging ecology and diet of gentoo penguins *Pygoscelis papua* at South Georgia and an assessment of their winter prey consumption. Ibis 133:3-13

Xavier JC, Croxall JP, Trathan PN, Wood AG 2003 Feeding strategies and diets of breeding grey-headed and wandering albatrosses at South Georgia. Marine Biology 143:221-232
