# Supplementary material for: At-Sea Distribution and Prey Selection of Antarctic Petrels and Commercial Krill Fisheries
Source: PLoS One. 2016 Aug 17;11(8):e0156968. doi: 10.1371/journal.pone.0156968 (PMC4988635; doi:10.1371/journal.pone.0156968)
Supplement: S1 Text — (DOCX) [file pone.0156968.s006.docx]

***S2 Text***. *Retrieval rate of GPS loggers deployed on Antarctic petrels in period 2011-2014, Svarthamaren colony, Dronning Maud Land.*

GPS have been deployed in three consecutive summers (2011/12, 2012/13 and 2013/14) on Antarctic petrels and to our knowledge, this represented the first GPS deployments on that species. No information from other sites was available so that we initially deployed loggers following methods used with other procellariform species, i.e. taped on the back of the birds [e.g. [1](#_ENREF_1),[2](#_ENREF_2)].

Deployments on the back of the birds were not successful (whatever the position on the back) and only 15-20% of the loggers were retrieved (*Table 1*). Antarctic petrels were indeed not tolerant to any loggers deployed on their back whatever the size and weight of the loggers. An experience performed in January 2013 showed that even loggers <5 grams (dummy loggers) were rapidly lost within 1-2 days after deployment.

From January 2013 onwards, loggers were deployed on the tail of the birds (taped around 2 feathers; *Picture 1*) and retrieval rate increased drastically (up to 74% in season 2013/14; *Table 1*).


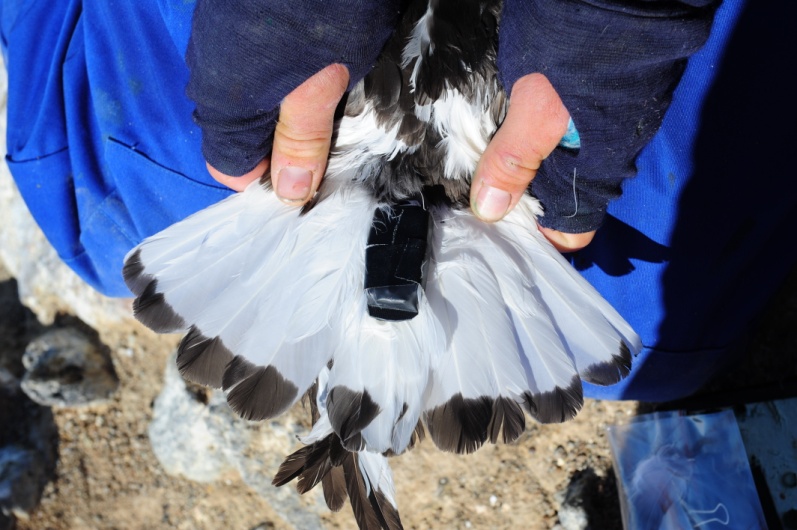


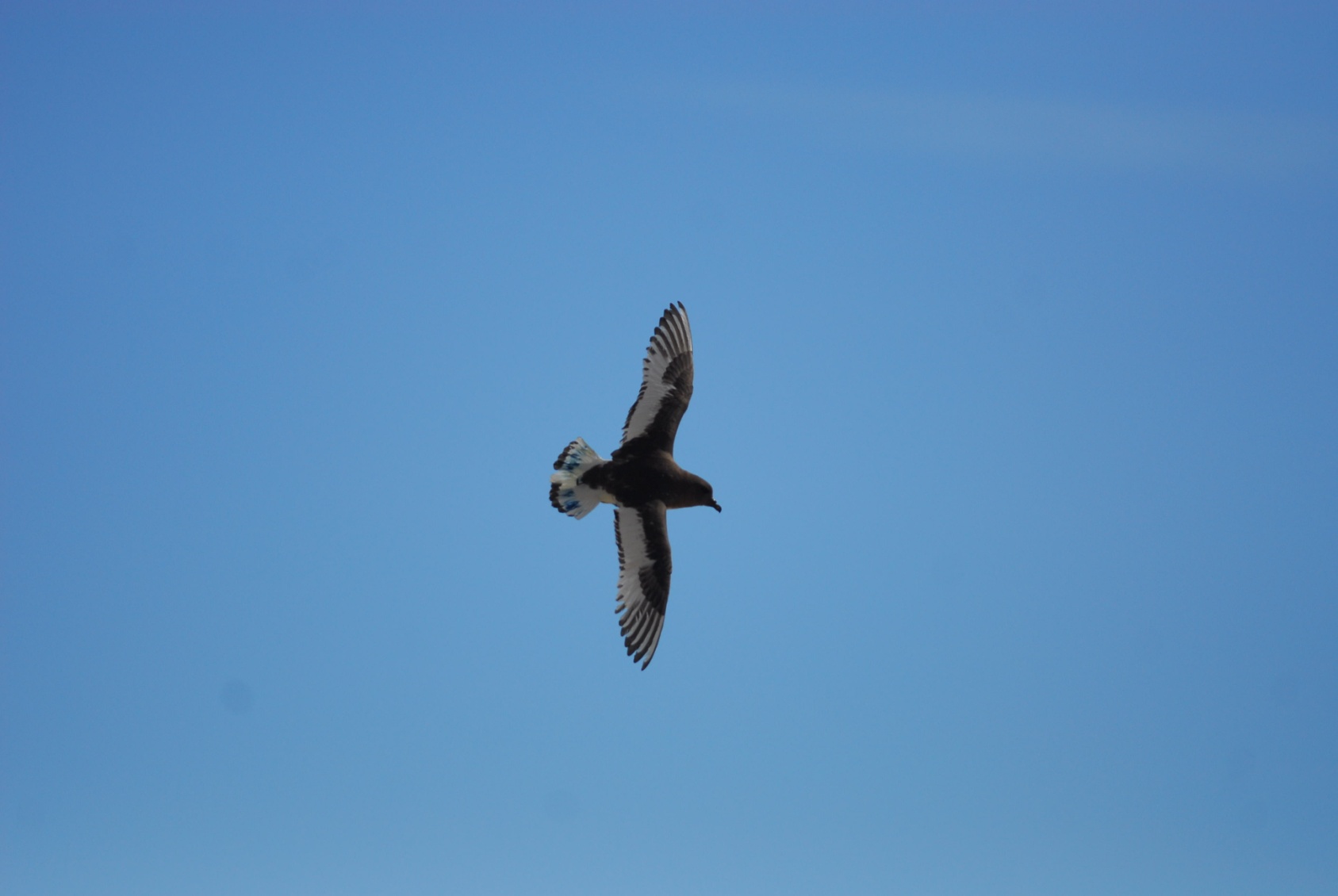


***Picture 1.*** *GPS logger deployed on the tail of an Antarctic petrel at Svarthamaren, Dronning Maud Land*

***Table 1.*** *Number of loggers deployed and retrieved on Antarctic petrels in three consecutive seasons using different deployment procedures. Number of retrieved loggers include all loggers, including those that did not work properly; this explains the difference between the total of loggers retrieved (n=138) and the total number of loggers used in our study (n=124).*

| ***Season*** | ***Deployment method*** | ***# deployed*** | ***# retrieved*** | ***% retrieved*** |
| --- | --- | --- | --- | --- |
| *2011-2012* | *Deployment on the back* | *129* | *19* | *15* |
| *2012-2013* | *Deployment on the back* | *30* | *6* | *20* |
|  | *Deployment on the tail* | *74* | *46* | *62* |
| *2013-2014* | *Deployment on the tail* | *91* | *67* | *74* |
|  |  |  |  |  |
| *All seasons* | *Deployment on the back* | *159* | *25* | *16* |
|  | *Deployment on the tail* | *165* | *113* | *68* |
| *All seasons* | *All deployment methods* | *324* | *138* | *43* |

***Literature cited***

1. Weimerskirch H, Delord K, Guitteaud A, Phillips RA, Pinet P (2015) Extreme variation in migration strategies between and within wandering albatross populations during their sabbatical year, and their fitness consequences. Scientific reports 5.

2. Patrick SC, Weimerskirch H (2014) Personality, Foraging and Fitness Consequences in a Long Lived Seabird. Plos One 9.
