# Supplementary material for: At-Sea Distribution and Prey Selection of Antarctic Petrels and Commercial Krill Fisheries
Source: PLoS One. 2016 Aug 17;11(8):e0156968. doi: 10.1371/journal.pone.0156968 (PMC4988635; doi:10.1371/journal.pone.0156968)
Supplement: S2 Text — (DOCX) [file pone.0156968.s007.docx]

***S3 Text***. *Foraging trip duration of control and experimental (i.e. fitted with a GPS logger) Antarctic petrels, Svarthamaren colony, Dronning Maud Land.*

In December 2013 and January 2014, a group of 13 control birds were monitored to estimate the duration of their foraging trips (11 birds in December and 2 in January, randomly chosen within our study plots). The duration of the foraging trips were similar for these control birds and for the birds fitted with a GPS logger (n=63 GPS birds in December 2013 and January 2014; p=0.66 for the effect of a two modality variable “control birds / GPS birds” (after adjusting for the date of the trip departure to take into account seasonal changes in trip duration; *Figure 1*).

*
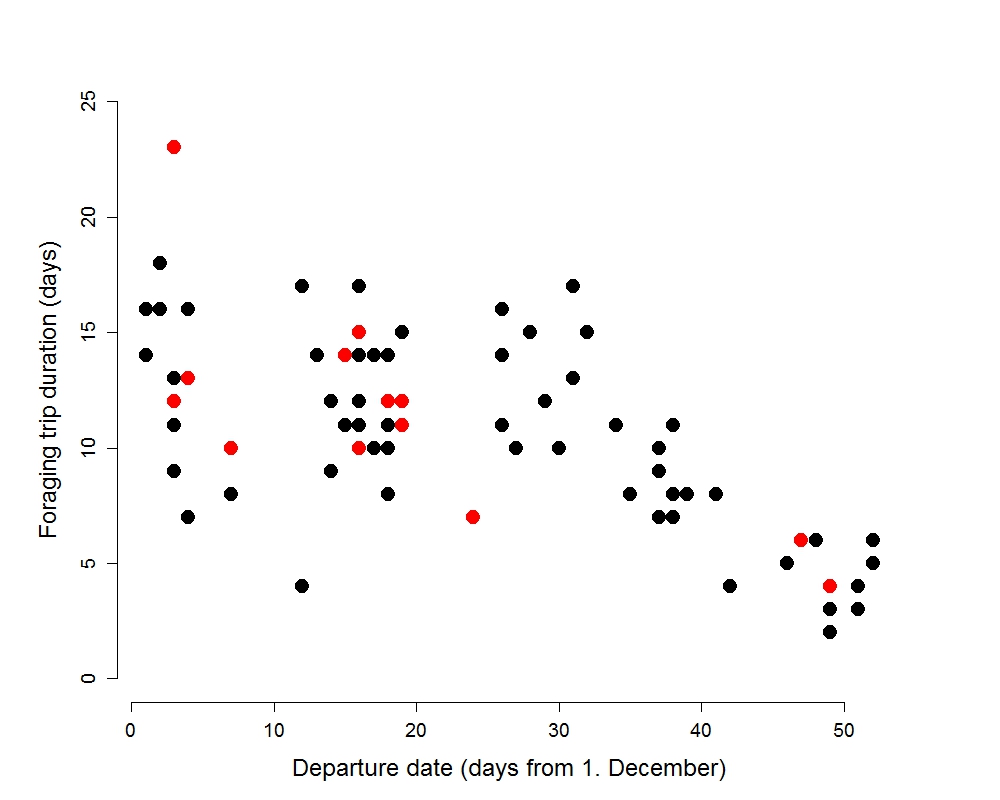
*

***Figure 1.*** *Duration of the foraging trips of Antarctic petrels. Red symbols represent control birds and black symbols birds fitted with an external GPS logger. Data are from December 2013 and January 2014 (Svarthamaren colony, Dronning Maud Land). The y-axis represent the number of days since December 1^st^.*
